# DDHTS-Net: dual-domain hierarchical texture supervision network for plant texture analysis

**DOI:** 10.3389/fpls.2026.1872418

**Published:** 2026-07-14

**Authors:** Bin Li, Ente Guo, Xiaochun Xu, Q. M. Jonathan Wu

**Affiliations:** 1 Fujian Agriculture and Forestry University, College of Computer and Information Science, Fuzhou, Fujian, China; 2 Minjiang University, School of Computer and Big Data, Fuzhou, Fujian, China; 3 Department of Electrical and Computer Engineering, University of Windsor, Windsor, ON, Canada

**Keywords:** contrast descriptor, deep feature maps, plant texture analysis, supervision unit, texture attribute

## Abstract

**Introduction:**

Texture is a fundamental low-level visual attribute characterized by complex local patterns and spatial structures. Plant textures, in particular, exhibit subtle inter-class differences and substantial intra-class variability, making accurate recognition challenging. This difficulty is further increased by fine-grained local texture variations and imaging conditions such as scale changes, illumination variations, and viewpoint differences. Existing deep texture analysis models often rely on a single image domain or representation stream, limiting their ability to capture complementary cross-frequency cues and hierarchical abstraction differences required for fine-grained texture discrimination.

**Methods:**

To address this challenge, this study proposes a dual-domain hierarchical texture supervision network, DDHTS-Net, designed to capture and model complex texture attributes across multiple domains and hierarchical levels. In DDHTS-Net, four channels with different granularity levels are constructed using the Undecimated Wavelet Transform. The network integrates an intra-channel hierarchical supervision unit and an inter-channel granularity-level supervision unit. The intra-channel unit treats deep feature maps from different layers as experts with distinct levels of abstract knowledge, enabling higher-level experts to guide lower-level counterparts in discovering latent and critical texture attributes. The inter-channel unit uses experts at different granularity levels within the same abstraction hierarchy to capture distinct spatial frequency characteristics and further supervise the latent texture features in each layer of the original image channel.

**Results:**

Extensive experiments on two plant classification datasets and two benchmark texture datasets demonstrate that DDHTS-Net consistently achieves superior classification performance compared with existing state-of-the-art methods.

**Discussion:**

These results indicate that integrating dual-domain representations with hierarchical texture supervision can effectively enhance fine-grained texture discrimination. The proposed DDHTS-Net provides an effective framework for modeling complex texture attributes in plant recognition and general texture classification tasks.

## 1 Introduction

Textures (He and Wang, 1990) are ubiquitous across the surfaces of nearly all objects. As a key attribute for object description, texture features provide critical information for a wide range of visual tasks, including material recognition (Bell et al., 2015), medical imaging analysis (Ghalati et al., 2021), remote sensing (Peng et al., 2022), and agricultural monitoring (Fritz et al., 2019). Consequently, the effective extraction of texture features from raw images has become a fundamental challenge in image processing and computer vision. Although humans can readily perceive and interpret the complex patterns inherent in visual textures and effectively discriminate contrast across different spatial frequency bands to capture fine-grained cues, replicating this capability in intelligent systems remains highly challenging. In practical applications, texture images often exhibit substantial intra-class variability and minimal inter-class differences due to variations in illumination, scale, rotation, and viewpoint, thereby posing significant challenges to texture classification and recognition.

Plant textures, which originate from the intricate arrangement of epidermal cells, venation patterns, trichomes, and other surface microstructures, represent a highly complex form of texture and have emerged as a discriminative source of information in visual plant classification tasks. In plant taxonomy, traditional attributes such as color, shape, or size are often insufficient for accurately distinguishing visually similar species. Conversely, fine-grained texture features (Boudra et al., 2022; Valarmathi et al., 2021) can capture subtle variations present in leaf surfaces, bark patterns, and floral structures, thereby enabling precise species identification. Advanced texture analysis techniques have demonstrated the capability to differentiate species within the same genus and even identify distinct cultivars exhibiting minimal morphological differences. In plant pathology (Moupojou et al., 2023; Paymode and Malode, 2022), pathological conditions frequently induce characteristic alterations in the textures of plant organs, including increased roughness, mottling, and irregular surface deformations. Quantifying these texture variations facilitates the discrimination between healthy and diseased plants and enables the assessment of infection progression and severity. Therefore, texture analysis plays an important role in early plant disease diagnosis, precision treatment, agricultural intelligent robotics (Li et al., 2022a; Chen et al., 2024b), and health monitoring, serving as a key technology in agricultural production and ecological research. Although plant texture analysis has significant application potential, due to the high diversity and complexity of plant textures, it still faces numerous challenges in both research and practical applications across fields such as botany, agricultural intelligent robotics (Li et al., 2022b; Chen et al., 2025), and environmental monitoring.

To extract effective texture features, researchers have developed a variety of methods to address challenges in texture classification and recognition. Early studies primarily relied on handcrafted features, such as Local Binary Patterns (LBP) (Ojala et al., 2002), wavelet transforms (Arivazhagan and Ganesan, 2003), fractal analysis (Xu et al., 2009), and bag-of-words models (Lima et al., 2019). Although these methods achieved favorable performance under controlled conditions, they often lacked robustness and generalization capability in complex real-world environments. With the rapid advancement of deep learning techniques, convolutional neural networks (CNNs) (Li et al., 2021; Gu et al., 2018) have demonstrated significant advantages in feature extraction. Through multilayer stacking, CNNs can automatically learn discriminative features directly from raw images, reducing reliance on expert-designed features and exhibiting strong cross-task transferability. In recent years, numerous deep learning-based methods have been proposed to enhance texture representation, achieving remarkable success in automatic feature extraction and classification tasks. Nevertheless, despite these advances, existing approaches still face difficulties in effectively modeling complex texture attributes, motivating further exploration of more sophisticated and efficient texture classification frameworks.

Local Binary Patterns (LBP) and their variants (Ojala et al., 2002; Hadid et al., 2015) represent some of the most successful handcrafted texture descriptors. By encoding the sign of local intensity differences within a neighborhood, LBP effectively captures fine-grained texture variations. Benefiting from the inherent advantages of binary operations and rotation-invariant uniform encoding, LBP exhibits strong robustness to illumination variations and rotational transformations. As a result, LBP has been extensively applied across a wide range of texture analysis domains, including remote sensing (Wang et al., 2022), face recognition (Huang et al., 2011), agricultural inspection (Singh et al., 2010), and scene understanding (Abidin et al., 2018). Nevertheless, despite their effectiveness in capturing texture-specific attributes, these handcrafted features heavily depend on expert knowledge and exhibit limited generalization ability, thereby significantly restricting their cross-domain applicability.

In recent years, deep learning models, particularly convolutional neural networks (CNNs), have demonstrated significant technical advantages in image classification. Unlike traditional handcrafted feature methods, CNNs are capable of automatically learning hierarchical feature representations from raw pixel data, enabling effective modeling of complex and abstract patterns without reliance on expert knowledge. However, conventional CNN architectures primarily focus on high-level semantic features, exhibiting limited sensitivity to fine-grained texture attributes and poor adaptability to scale variations. These limitations are further exacerbated when addressing substantial intra-class variations commonly observed in texture categories. As a result, the learning of deep texture features has emerged as a critical and active research direction in texture classification. To overcome these limitations, various approaches have been proposed. Liu et al. (2019) introduce a GANet architecture, which addresses the challenge of extreme scale variations in texture images. By incorporating a genetic algorithm, GANet dynamically adapts the filters in its hidden layers during training, thereby enhancing the network’s capability to capture semantic texture patterns across multiple scales. Scabini et al. (2020) present a directed complex network specifically designed for color texture analysis, termed the Spatio-Spectral Network (SSN), which effectively models topological features within a multiscale framework based on radially symmetric neighborhoods. In another work, Mao et al. (2021) propose a Deep Residual Pooling Network that integrates a residual encoding module with an aggregation module, forming a residual pooling layer, effectively reducing feature dimensionality while maintaining high recognition performance. Beyond addressing complex imaging challenges, recent studies have also explored the integration of multi-level texture features. Tao et al. (2021) propose the Wavelet Multi-level Attention Capsule Network (WMACapsNet), which jointly leverages spatial domain features, frequency domain features, and their interdependencies to significantly enhance texture classification performance. Moreover, Chang et al. (2024) introduce a Cellular Neural Network (CellNN) with a recurrent convolutional structure, enabling the cross-domain fusion of LBP features with image contours and other complementary representations, thus substantially improving texture feature modeling. Additionally, Florindo et al. (2024) propose ELMP-Net, which is based on the successive application of a local transform. By constructing a two-layer mapping structure, ELMP-Net effectively captures local texture attributes and demonstrates strong performance on benchmark texture datasets as well as in plant species identification tasks.

Another line of research explores the underlying relationships among multiple attributes to construct more effective texture representations. To capture the inherent structural properties of texture images, Zhai et al. (2020) propose a Deep Structure-Revealed Network (DSR-Net), which models the spatial dependencies among fundamental texture primitives. The resulting structural representation significantly enhances texture recognition performance in real-world scenarios. Building upon the idea of modeling interactions between feature elements, Dong et al. (2020) introduce Pair-Wise Difference Pooling-Based Bilinear Convolutional Neural Networks. By capturing pairwise relationships among feature maps extracted from a convolutional layer of a pre-trained CNN and encoding the differences between each pair, this approach enables the effective extraction of discriminative texture features. To address the challenge of capturing inter-scale relationships in texture analysis, Boudra et al. (2022) combine multi-scale statistical macro binary patterns (ms-SMBP) with a CNN framework. By modeling the correlations across different scale hierarchies and their adaptive statistical prototypes, the proposed method achieves effective automated classification of tree trunk species. Similarly, Mohan and Peeples (2024) focus on improving pooling operations in CNNs for texture tasks, proposing a novel lacunarity pooling layer designed to capture intricate spatial patterns by explicitly modeling the spatial arrangement of pixel intensities and feature values. This innovative pooling strategy has demonstrated superior performance, particularly in agricultural image analysis applications. Furthermore, several studies have explored the modeling of directional and hierarchical relationships among visual attributes. Dai et al. (2024) design a multi-level Deep Information Feature Fusion Network (DFN) to enhance the localization of infected regions in plant disease detection. In the domain of plant leaf identification, Wu et al. (2023) addresses the problem of large intra-class variation and small inter-class differences by integrating complementary shape features with convolutional features, demonstrating significant advantages in fine-grained plant classification tasks. For texture datasets characterized by high intra-class similarity, Sakthipriya et al. (2024) introduce the Texture Strip Dual Attention Network (TSDA-Net), which effectively captures key discriminative features among visually similar texture subtypes, thereby enhancing the network’s ability to distinguish subtle differences. Ali et al. (2024) integrated multiple deep learning architectures and employed a class-weighting algorithm to balance the data, thereby enhancing the performance of plant disease classification. Moreover, to mitigate the issue of CNN architectures overly relying on high-level features while neglecting low- and mid-level information, Park et al. (2024) introduce a Feature Retention Module (FRM) that effectively leverages texture attributes extracted across all convolutional layers, resulting in a substantial improvement in classification performance. Finally, Chen et al. (2024a) propose a deep tracing pattern encoding method, which constructs a highly discriminative and robust global representation by exploiting low-level visual cues from shallow layers as well as cross-layer feature interactions within the network.

Overall, although existing methods have provided effective solutions for deep texture analysis, they still exhibit several key limitations. Handcrafted features offer strong interpretability but limited generalization ability; conventional CNN-based methods tend to focus on high-level semantic representations and are insufficiently sensitive to fine-grained texture details and scale variations; and multi-scale or multi-layer approaches, while partially alleviating these issues, generally lack explicit cross-frequency and cross-channel supervision mechanisms. To address these limitations, this paper proposes a dual-domain hierarchical texture supervision network (DDHTS-Net). Specifically, the proposed method constructs multi-frequency channels using the Undecimated Wavelet Transform (UWT), and introduces two complementary supervision mechanisms: the intra-channel hierarchical supervision unit (IntraHSU), which models cross-layer feature dependencies, and the inter-channel granularity-level supervision unit (InterGSU), which captures cross-frequency interactions. Through this design, the proposed method effectively bridges the above gaps and enables more comprehensive modeling of complex plant texture attributes.

To tackle these challenges, this paper introduces a dual-domain hierarchical texture supervision network (DDHTS-Net). The key contributions of this study are summarized as follows:

1. The Undecimated Wavelet Transform (UWT) is employed to construct four channels with distinct spatial frequency bandwidths, including the original image. The high-frequency channels focus on capturing complex local texture details, whereas the low-frequency channels extract global structural and contour information. This frequency band partitioning emulates the human visual system’s processing of texture information, thereby enhancing the generalization capability of DDHTS-Net in complex texture classification tasks.
2. Intra-channel hierarchical supervision unit treats deep feature maps from different layers as experts possessing varying levels of abstract knowledge. A simple yet efficient difference-based binary supervision mechanism, based on the differences between adjacent deep feature maps, is employed to enable higher-level experts to guide their lower-level counterparts. This strategy facilitates the discovery of latent and critical texture attributes.
3. In the inter-channel granularity-level supervision unit, experts at different granularity levels within the same abstraction hierarchy exhibit distinct spatial frequency characteristics. At each abstraction level, a difference-based inter-channel binary supervision mechanism is employed, enabling experts from different granularity levels to further supervise and guide the knowledge embedded in the corresponding layers of the original image channel. This strategy facilitates the effective capture and perception of complex cross-domain texture attributes.
4. To validate the effectiveness of DDHTS-Net, extensive experiments were performed on two plant classification datasets and two benchmark texture datasets. The experimental results confirm that DDHTS-Net consistently outperforms existing state-of-the-art methods in both texture image and plant leaf classification tasks.

The remainder of this paper is organized as follows: Section 2 presents the materials and methods. Experimental evaluations are provided in Section 3. Finally, Section 4 concludes the paper.

## 2 Materials and methods

### 2.1 Multi-band contrast descriptor

It is well established that the human visual system exhibits a highly sophisticated capacity for processing spatial information, with contrast perception across different spatial frequency bands serving as one of its fundamental perceptual mechanisms. Image components at distinct frequency bands typically correspond to visual information at varying levels of granularity: low-frequency components capture large-scale contours and structural layouts, while high-frequency components reflect fine details, edges, and texture patterns. The variation in contrast across these frequency bands enables the human visual system to effectively recognize objects, perceive intricate structures, and discern subtle texture variations. As outlined above, this fine-grained perceptual mechanism provides a robust theoretical foundation and valuable inspiration for the design of algorithms in image processing and computer vision.

**Figure 1:**
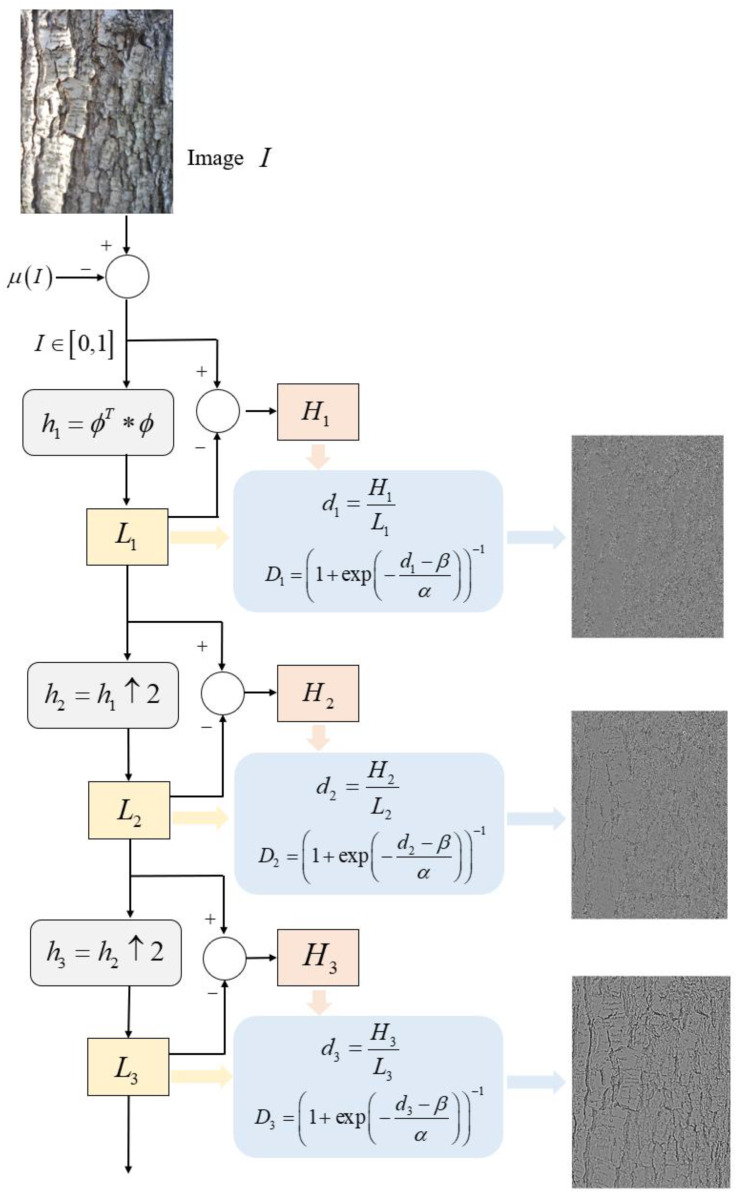
The illustration of multi-band contrast descriptor.

In 2017, Syed et al. (2017) introduced the Local Contrast Descriptor (LCD), which enables dense, pixel-level contrast representation across multiple spatial frequency scales, demonstrating notable robustness against noise and illumination variations. Building upon the theoretical foundation established by LCD, we designed the multi-band contrast descriptor, as illustrated in **Figure 1**, to introduce three additional processing channels to DDHTS-Net beyond the original texture image.

To analyze texture information across different spatial frequency bands, the Undecimated Wavelet Transform (UWT) (Starck et al., 2007) is first applied to decompose the input texture image into multiple spatial frequency components. Let the image I be represented as the sum of its low-frequency, mid-frequency, and high-frequency components, denoted as $I_{L}$, $I_{M}$, and $I_{H}$, respectively. Then, the image can be expressed as shown in Equation (1):

(1) $$I(i,j)=I_{L}(i,j)+I_{M}(i,j)+I_{H}(i,j)$$

Using the isotropic Undecimated Wavelet Transform (IUWT), let $H_{k}(i,j)$ and $L_{k}(i,j)$ denote the high-frequency and low-frequency components at spatial scale kand spatial location (i,j), respectively. The corresponding IUWT operations are given in Equations (2)–(4):

(2) $$H_{k}(i,j)=L_{k-1}(i,j)-L_{k}(i,j)$$

(3) $$L_{k}(i,j)=L_{k-1}(i,j)*h_{j}$$

(4) $$h_{k}=h_{k-1}↑2$$

where $h_{k}$ represents an isotropic low-pass filter at scale $k^{th}$ (i.e., the up-sampled version of the filter at that scale ${\left(k-1\right)}^{th}$).

It is worth noting that the original image corresponds to $L_{0}$. The two-dimensional filter $h_{1}$ is constructed using separable and symmetric coefficients through convolution. The mathematical expression is given in Equation (5):

(5) $$h_{1}=\phi^{T}*\phi =\frac{1}{16}(\begin{array}{ccc} 1 & 2 & 1\\ 2 & 4 & 2\\ 1 & 2 & 1 \end{array})$$

where $\phi =\frac{1}{4}[1\;2\;1]$. Due to the symmetry and isotropy of the filter, the IUWT exhibits robustness to image rotation. The local contrast at spatial location (i,j) is defined in Equation (6):

(6) $$d_{k}(i,j)=\frac{H_{k}(i,j)}{L_{k}(i,j)}$$

The above equation estimates the local contrast within each spatial frequency band, which is determined by the local energy and the background luminance at the corresponding spatial scale. However, when $H_{k}(i,j)$ is much larger or much smaller than $L_{k}(i,j)$, the value of the local contrast $d_{k}(i,j)$increases significantly. To mitigate this effect, Syed et al. (2007) proposed applying a sigmoidal intensity mapping to the local contrast, defined in Equation (7):

(7) $$D_{k}(i,j)={\left(1+\exp(-\frac{d_{k}(i,j)-\beta }{\alpha })\right)}^{-1}$$

where β denotes the mean of $d_{k}$, and α is a compression control factor, which is empirically set to the standard deviation of $d_{k}$.

**Figure 1** illustrates the construction process of the multi-band contrast descriptor, which decomposes the input image into multiple spatial frequency bands using the Undecimated Wavelet Transform (UWT), and then computes contrast representations across different frequency scales. Given an input image I, the process first performs intensity normalization μ(I) to ensure numerical stability. The normalized image is then decomposed into a hierarchical set of frequency components through iterative filtering operations. At each scale k, the low-frequency component $L_{k}$ and high-frequency component $H_{k}$ are generated using upsampled filtering and subtraction operations. This results in a multi-scale pyramid structure, where $L_{k}$ preserves coarse structural information while $H_{k}$ captures fine-grained texture details such as edges and local intensity variations. Next, a local contrast representation $d_{k}$ is computed at each scale by measuring the ratio between high-frequency and low-frequency responses. A sigmoid-based normalization mapping is then introduced to enhance the model’s robustness against extreme contrast variations, yielding $D_{k}$. Finally, the outputs $D_{1}$, $D_{2}$,and $D_{3}$ correspond to different spatial frequency components (high-, mid-, and low-frequency bands), jointly forming the multi-band contrast descriptor and providing complementary multi-scale texture information. Overall, this design mimics the perceptual mechanism of the human visual system for multi-scale contrast variations, thereby significantly improving robustness and discriminative capability in complex texture analysis tasks.

### 2.2 Normalized deep feature maps

Convolutional neural networks (CNNs) progressively extract feature representations from input images through successive convolutional operations and nonlinear transformations. This hierarchical processing paradigm enables different layers of the network to capture features at varying levels of abstraction.

In the present study, the deep feature maps at each layer are conceptualized as domain experts possessing knowledge at distinct abstraction levels. Specifically, shallow experts focus on low-level local visual attributes and excel at identifying fine-grained texture details. Intermediate experts specialize in modeling local structural patterns and integrating low-level features through combination and reconstruction, exhibiting a certain degree of contextual awareness. Deep experts capture high-level semantic information and demonstrate superior abstraction and generalization capabilities. This expert analogy provides a conceptual foundation for understanding the collaborative mechanisms of the intra-channel hierarchical supervision unit and the inter-channel granularity-level supervision unit proposed in subsequent sections of this paper.

To effectively process the feature maps from different layers of the deep neural network, this section adopts a down-sampling and average reduction strategy to standardize them. Taking AlexNet as an example, deep feature maps are extracted from four convolutional layers—conv2, conv3, conv4, and conv5—using input images resized to 227 × 227 × 3.

**Figure 2:**
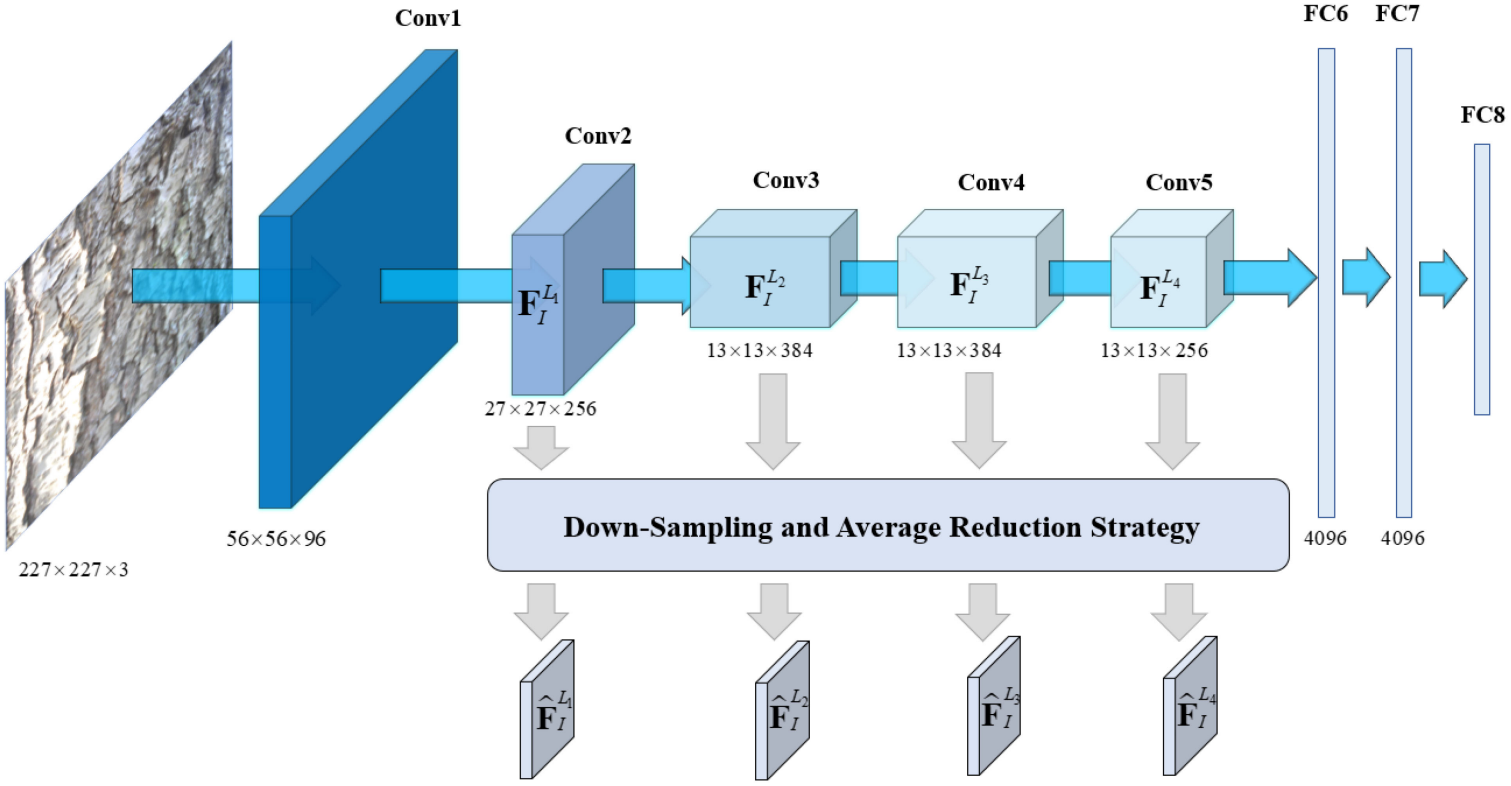
The illustration of normalized feature maps.

As shown in **Figure 2**, after feeding the original image I into the backbone network, the original deep feature maps extracted from conv2, conv3, conv4, and conv5 are denoted as $F_{I}^{L_{1}}$, $F_{I}^{L_{2}}$, $F_{I}^{L_{3}}$, and $F_{I}^{L_{4}}$, respectively. These feature maps are subsequently standardized using the down-sampling and average reduction strategy, resulting in the normalized feature maps ${\hat{F}}_{I}^{L_{1}}$, ${\hat{F}}_{I}^{L_{2}}$, ${\hat{F}}_{I}^{L_{3}}$, and ${\hat{F}}_{I}^{L_{4}}$.We represent the set of normalized feature maps as: ${\text{ℱ}}_{I}=\{{\hat{F}}_{I}^{L_{i}}\in {\mathbb{R}}^{H\times W\times C}|i=1,2,\ldots N\}$。Taking AlexNet as an example, N=4. In addition, the multi-band contrast descriptors generated from different frequency bands in **Figure 1** are also fed into the backbone network to obtain the corresponding multi-band contrast-enhanced deep feature map sets, denoted as ${\text{ℱ}}_{D_{k}}={\hat{F}}_{D_{k}}^{L_{i}}\in {\mathbb{R}}^{H\times W\times C}|i=1,2,\ldots N\},k=1,2,3$.

### 2.3 Intra-channel hierarchical supervision unit

As outlined above, experts from different layers of the deep neural network encode texture features at distinct levels of abstraction. The differences between adjacent-layer experts encapsulate a wealth of latent and critical texture attributes. To enable supervisory collaboration among these adjacent-layer experts, this study introduces the intra-channel hierarchical supervision unit (IntraHSU).

Existing theories, such as Local Binary Patterns (LBP) and its variants, have demonstrated that local difference information serves as a crucial cue for capturing texture characteristics. Motivated by this insight, the concept of local difference modeling is incorporated into deep feature representations to enable collaborative supervision among adjacent-layer experts, thereby extracting latent and critical texture attributes. Specifically, a difference-based binary supervision mechanism is proposed, wherein higher-level feature maps supervise lower-level feature maps through binary encoding of their differences. This strategy facilitates interactive learning of key texture attributes across adjacent layers while preserving the integrity of texture information at various depths. As a result, the proposed intra-channel hierarchical supervision unit (IntraHSU) enables mutual supervision and collaborative learning between feature maps of successive depths, thereby enhancing the network’s capacity to capture and represent complex texture patterns.

**Figure 3:**
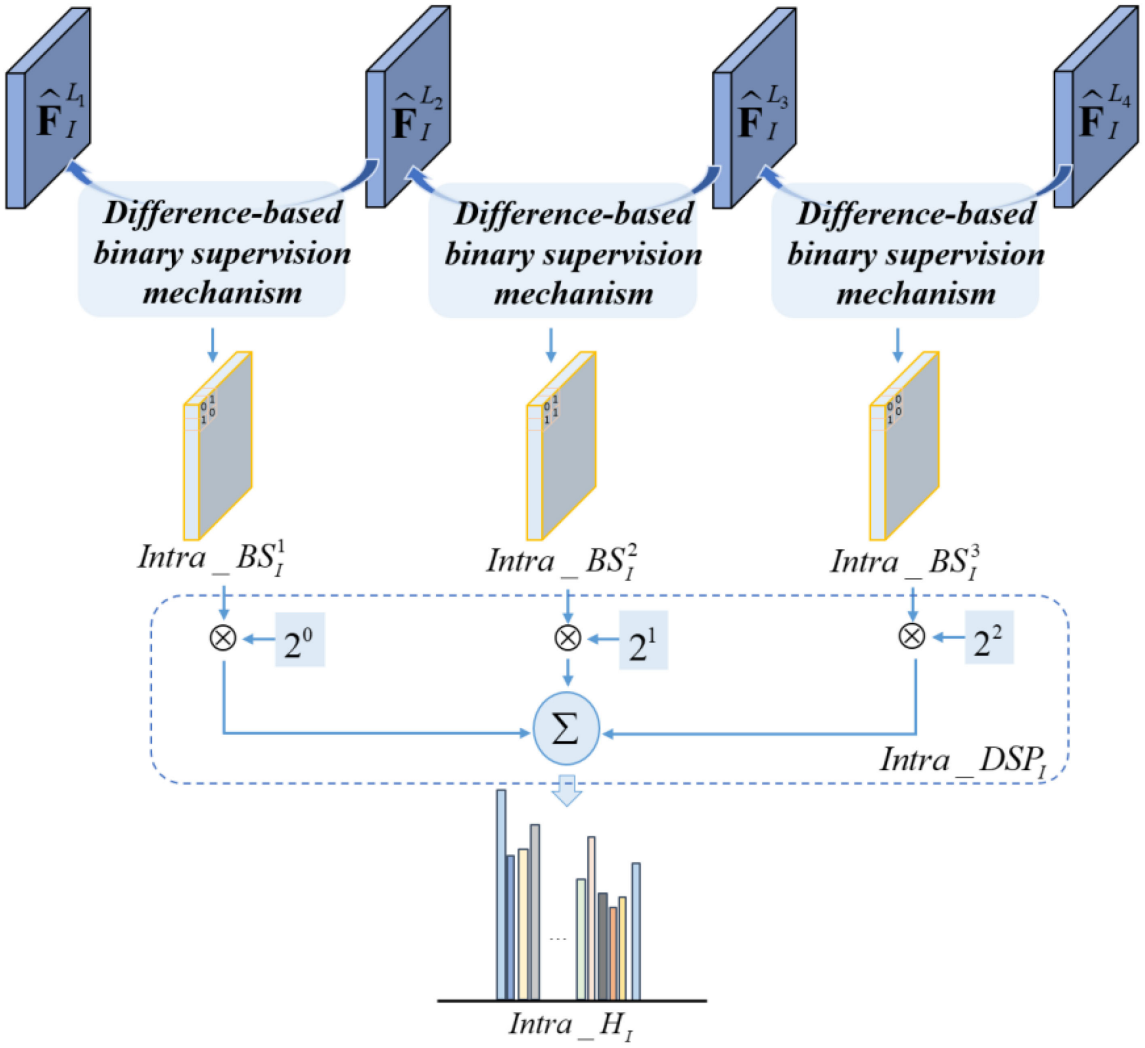
The illustration of intra-channel hierarchical supervision unit (IntraHSU).

As illustrated in **Figure 3**, for the normalized feature maps of original image channel ${\text{ℱ}}_{I}={\hat{F}}_{\text{I}}^{{\text{L}}_{\text{i}}}\in {\mathbb{R}}^{H\times W\times C}|i=1,2,\ldots N\}$, the difference-based binary supervision mechanism is applied to adjacent deep feature maps, which can be mathematically expressed in Equation (8):

(8) $$Intra\_BS_{I}^{i-1}=sign({\hat{F}}_{I}^{L_{i}}-{\hat{F}}_{I}^{L_{i-1}}),i=1,2,\cdots ,N$$

where $Intra\_BS_{I}^{i-1}$ is the intra-channel binary supervision mapping. When operations are applied to N feature mapping layers, N−1 intra-channel binary supervision mappings are produced, each capturing the differences between successive layers to facilitate hierarchical supervision. sign(·) denotes the sign function, defined in Equation (9):

(9) $$sign(x)=\{\begin{array}{c} 1,\;x\geq 0\\ 0,\;x<0 \end{array}$$

As illustrated in Equation 8, the difference-based binary supervision mechanism encodes the signs of the differences between adjacent-layer experts, thereby enabling effective supervision and guidance of lower-level texture attributes by higher-level representations. Concurrently, this mechanism promotes the interaction and collaborative learning of texture information across adjacent feature mapping layers. The binary encoding approach is inspired by the core principles of the binary pattern family, offering both simplicity and computational efficiency while aligning with the natural distribution patterns of texture attributes observed in real-world images.

To effectively integrate the intra-channel binary supervision mapping, this paper re-encodes them into a new (N−1)−bit binary pattern mapping, referred to as the intra-channel deep supervision pattern. As shown in **Figure 3**, this pattern is subsequently converted into its decimal form, and a statistical histogram is computed to derive the final feature vector, denoted in Equation (10)–(12):

(10) $$Intra\_DSP_{I}=Intra\_BS_{I}^{1}\times 2^{0}+Intra\_BS_{I}^{2}\times 2^{1}+\cdots +Intra\_BS_{I}^{N-1}\times 2^{N-2}$$

(11) $$Intra\_H_{I}(h)=\sum_{i=1}^{H}\sum_{j=1}^{W}\delta (Intra\_DSP(i,j)-h),h=0,1,\cdots ,2^{N-1}-1$$

(12) $$\delta (x-c)=\{\begin{array}{c} 1,\;x=c\;\\ 0,\;otherwise \end{array}$$

Building on the theory, for the normalized feature maps ${\text{ℱ}}_{I}$, we can obtain the feature histogram $Intra\_H_{I}$ of the $Intra\_DSP_{I}$. To capture the rich texture information carried by different contrast maps at varying spatial scales, we apply the difference-based binary supervision mechanism to the multi-band contrast-enhanced deep feature map sets ${\text{ℱ}}_{D_{k}},k=1,2,3$, and obtain the corresponding feature vectors $Intra\_H_{D_{k}},k=1,2,3$ of $Intra\_DSP_{D_{k}}$.

As illustrated in **Figure 3**, for the Nexperts within original image channel, the dimensionality $Intra\_H_{I}$ of the resulting feature is $2^{N-1}$. Similarly, the multi-band contrast descriptors $D_{k},k=1,2,3$ are fed into the respective network channels to obtain $Intra\_H_{D_{k}}$​, each with a feature dimensionality of $2^{N-1}$. Finally, $Intra\_H_{I}$ and $Intra\_H_{D_{k}}$​ are concatenated to generate the output histogram of the intra-channel hierarchical supervision unit, denoted as Intra_H, with a total feature dimensionality of $4\times 2^{N-1}$, described in Equation (13).

(13) $$Intra\_H=[Intra\_H_{I},Intra\_H_{D_{1}},Intra\_H_{D_{2}},Intra\_H_{D_{3}}]$$

Accordingly, within each channel, each supervision map captures the critical texture attributes between adjacent deep feature layers, thereby enabling the fine-grained extraction and enhancement of texture features across different levels of abstraction. The histogram Intra_H thus integrates the comprehensive texture information derived from the original image channel and the three different granularity levels channels.

Based on the above theoretical analysis, it is worth clarifying that “hierarchical” refers to the fact that feature maps at different convolutional depths correspond to different levels of abstraction: shallow layers focus on edges, textures, and local details; intermediate layers capture local structural information; and deeper layers possess stronger semantic and global representation capabilities. “Supervision” does not refer to additional class labels or an extra loss function. Instead, it denotes feature-level guidance achieved by binarizing the signs of differences between normalized adjacent-layer feature maps, enabling higher-level feature maps to guide the selection and statistical encoding of lower-level texture information through structured difference patterns.

### 2.4 Inter-channel granularity-level supervision unit

To achieve a comprehensive understanding and representation of the complex properties exhibited by natural textures, this study introduces an inter-channel granularity-level supervision unit (InterGSU). Specifically, the multi-band contrast descriptors $D_{k},k=1,2,3$ generated in **Figure 1** are input into a deep neural network, producing deep feature experts at different granularity levels. It can thus be observed that experts at the same abstraction level but different granularity levels possess distinct spatial frequency characteristics. In this section, a difference-based inter-channel binary supervision mechanism is employed, enabling experts at different granularity levels to further supervise and guide the knowledge embedded in the corresponding layers of the original image channel. This strategy facilitates the effective capture and perception of complex cross-domain texture attributes.

The unit simultaneously inputs the original image I and multi-band contrast descriptors $D_{k},k=1,2,3$ into the deep neural network. Based on the down-sampling and average reduction strategy, we can derive the set of normalized original image feature maps ${\text{ℱ}}_{I}$, along with the corresponding multi-band contrast-enhanced deep feature map sets ${\text{ℱ}}_{D_{k}},k=1,2,3$. Consequently, feature maps from different layers across various networks capture a wide range of semantic and structural information, thus providing a more comprehensive and enriched understanding of texture attributes for the texture classification task. This approach facilitates the detailed analysis of texture at various levels, thereby enhancing classification performance.

**Figure 4:**
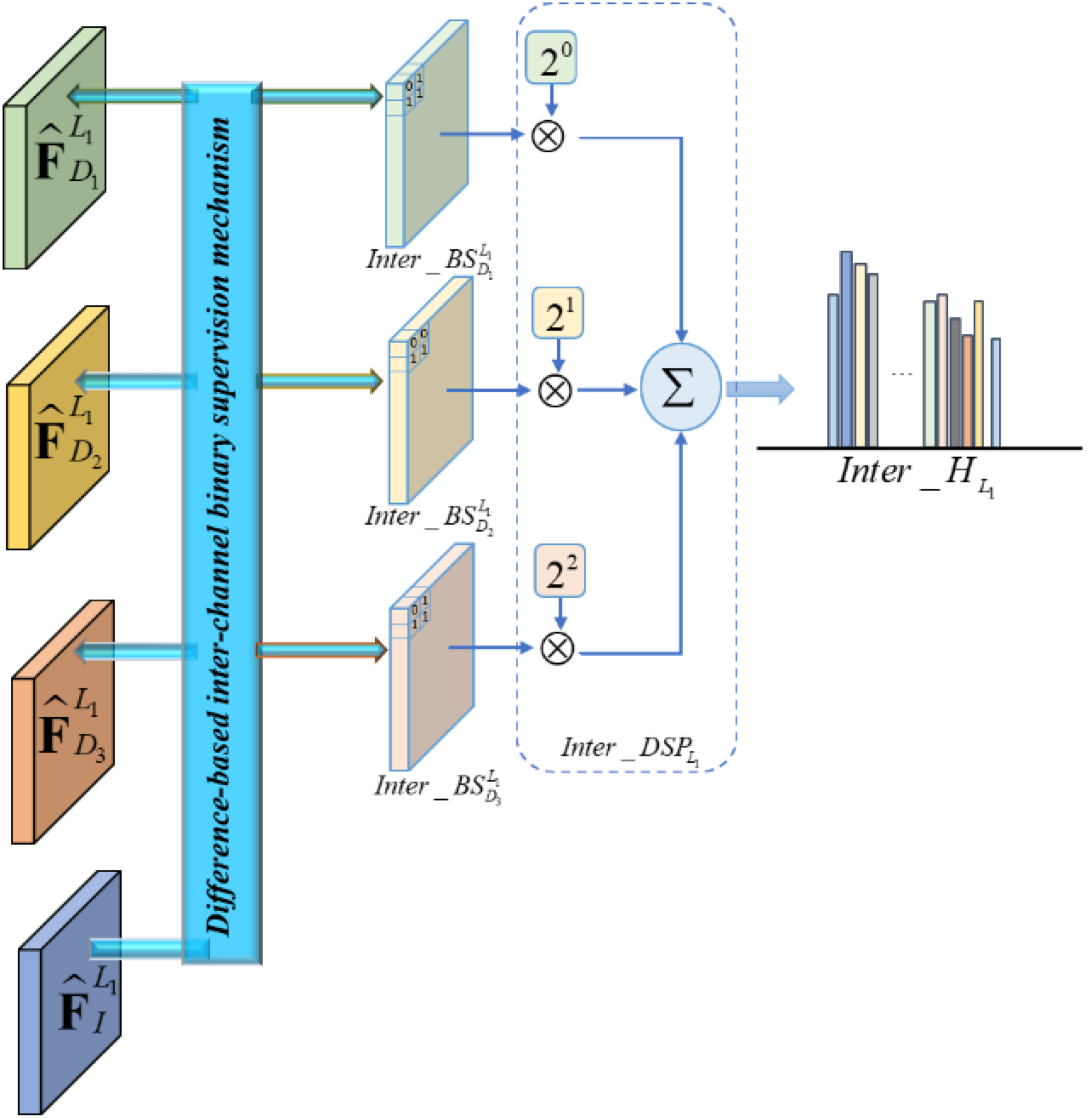
The illustration of inter-channel granularity-level supervision unit (InterGSU).

As illustrated in **Figure 4**, the difference-based inter-channel binary supervision mechanism is sequentially applied to ${\text{ℱ}}_{I}$ and ${\text{ℱ}}_{D_{k}},k=1,2,3$. For the layer $L_{i}$, this process results in the generation of the inter-channel binary supervision mapping $Inter\_BS_{D_{k}}^{i}$, which is mathematically defined in Equation (14).

(14) $$Inter\_BS_{D_{k}}^{Li}=sign({\hat{F}}_{D_{k}}^{L_{i}}-{\hat{F}}_{I}^{L_{i}}),i=1,2,\cdots ,N$$

where sign(·) is the sign function, defined in Equation 9.

As illustrated in Equation 14, the multi-band contrast-enhanced deep feature maps are used to supervise and guide the feature maps from the original image, facilitating effective interaction and complementary enhancement of the multi-band deep attributes across networks.

Similar to intra-channel hierarchical supervision unit, for layer $L_{i}$, the obtained $Inter\_BS_{D_{k}}^{i}$ are re-encoded into a new 3-bit binary pattern, termed the inter-channel deep supervision pattern. As shown in **Figure 4**, we convert this pattern into its decimal form and compute its statistical histogram to obtain the final feature vector, described in Equations (15) and (16):

(15) $$Inter\_DSP_{L_{i}}=Inter\_BS_{D_{1}}^{Li}\times 2^{0}+Inter\_BS_{D_{2}}^{Li}\times 2^{1}+Inter\_BS_{D_{k}}^{Li}\times 2^{2}$$

(16) $$Inter\_H_{L_{i}}(h)=\sum_{i=1}^{H}\sum_{j=1}^{W}\delta (Inter\_DSP_{L_{i}}(i,j)-h),h=0,1,\cdots ,7$$

where δ(·) is defined in Equation 12. As shown in Equation 16, we represent the histogram of $Inter\_DSP_{L_{i}}$ as $Inter\_H_{L_{i}}$. Thus, for layer $L_{i},i=1,2,\cdots ,N$, we obtain N corresponding intra-channel supervision histograms, and the dimensionality of each histogram is 8. By concatenating all the intra-channel supervision histograms $Inter\_H_{L_{i}},i=1,2,\cdots ,N$, the output histogram Inter_H of the inter-channel granularity-level supervision unit (InterGSU) is obtained, with a feature dimensionality of 8N, denoted in Equation (17):

(17) $$Inter\_H=[Inter\_H_{L_{1}},Inter\_H_{L_{2}},\cdots ,Inter\_H_{L_{N}}]$$

Equations 14 and 15 implement the interaction between different channels through binary encoding and re-encoding, thereby facilitating the complementary enhancement of texture attributes from multiple spatial frequency perspectives. This cross-channel synergy enhances the overall performance of the network in complex texture classification tasks. Furthermore, the fusion of deep texture attributes across multiple networks mitigates feature overfitting to any single network or frequency band, thereby improving the network’s generalization capability and its performance on unseen data.

### 2.5 Dual-domain hierarchical texture supervision network

Natural textures frequently exhibit a range of inherently contradictory properties, including regularity and irregularity, homogeneity and heterogeneity, locality and globality, stability and variability, as well as determinism and randomness. These complex and conflicting attributes not only underscore the intrinsic complexity of texture patterns found in natural scenes but also present substantial challenges for existing texture analysis methodologies. Accordingly, this study seeks to develop an effective modeling framework capable of capturing the multifaceted nature of textures, thereby enabling a comprehensive understanding and representation of their diverse characteristics.

**Figure 5:**
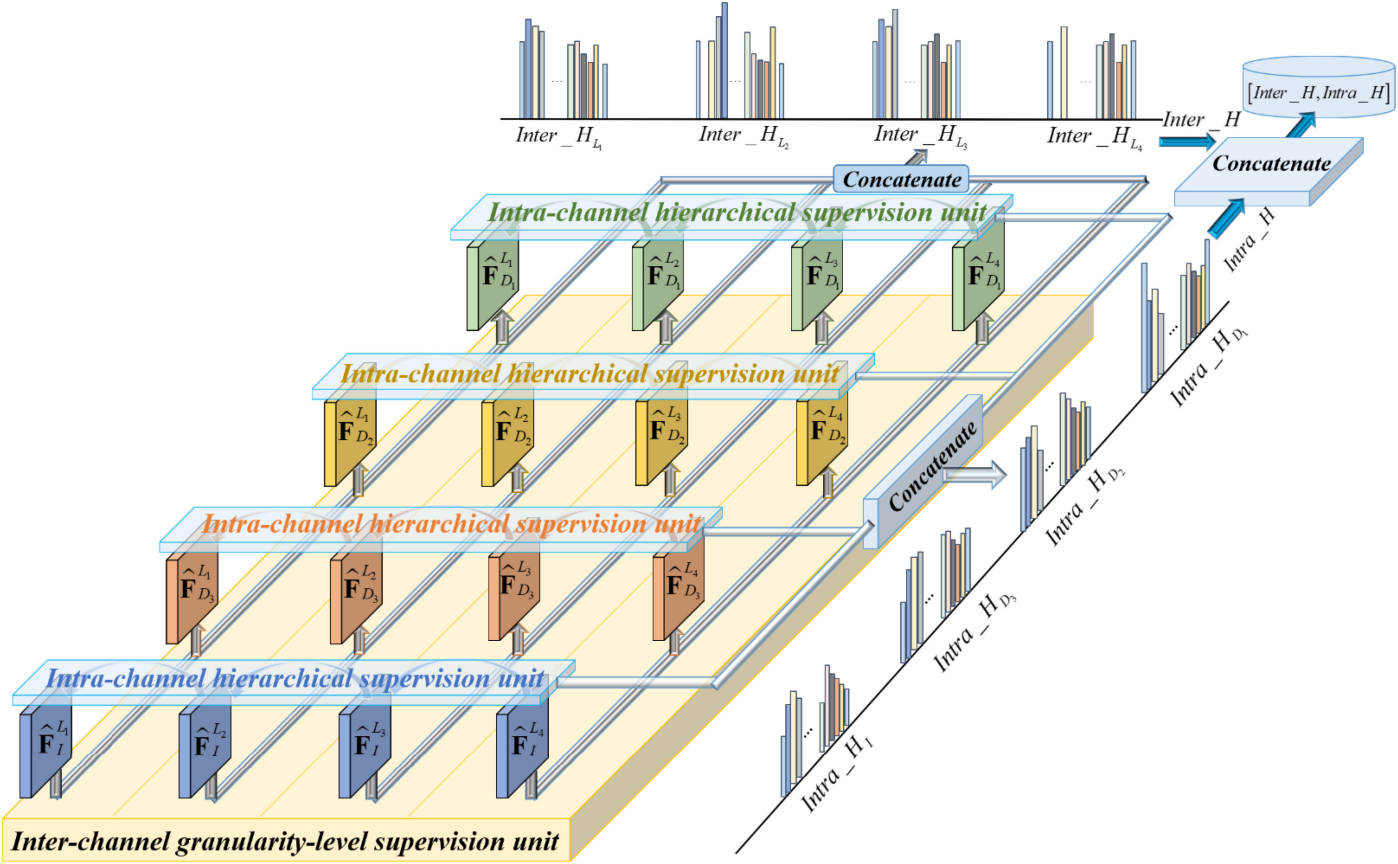
The illustration of dual-domain hierarchical texture supervision network (DDHTS-Net).

To effectively capture and model complex texture attributes across multiple domains and hierarchical levels, the Dual-Domain Hierarchical Texture Supervision Network (DDHTS-Net) is proposed in this section, as shown in **Figure 5**. Specifically, DDHTS-Net constructs a four-channel network that takes the original texture image and three multi-band contrast descriptors as inputs. It is noteworthy that the four-channel structure is not arbitrarily designed, but is determined by the construction principle of the proposed multi-band contrast descriptor. Specifically, one channel corresponds to the original image I, while the other three channels are derived from the multi-band contrast descriptors $D_{1}$, $D_{2}$, and $D_{3}$, which are generated based on the UWT decomposition and sigmoid-based contrast mapping. Theoretically, the original image channel preserves the complete spatial appearance information. In contrast, the high-, mid-, and low-frequency contrast channels respectively emphasize fine-grained details, local structural patterns, and coarser texture/contour information. Therefore, the four-channel architecture can be interpreted as a unified representation of the “original domain + three spatial frequency/granularity domains.” In **Figure 5**, each channel’s feature maps are depicted in a different color.

Deep feature maps from different convolutional layers within each channel are extracted and regarded as experts possessing varying levels of abstract knowledge. Within each channel, an intra-channel hierarchical supervision unit is implemented, employing a binary supervision mechanism based on the differences between adjacent deep feature maps. This mechanism enables higher-level experts to supervise and guide their lower-level counterparts in capturing latent and critical texture attributes. Across channels, an inter-channel granularity-level supervision unit is introduced, utilizing a difference-based binary supervision mechanism that allows experts from different granularity levels to further supervise and guide the knowledge embedded in the corresponding layers of the original image channel.

To achieve a comprehensive understanding and representation of texture attributes, as illustrated in **Figure 5**, the output histogram Intra_H of the intra-channel hierarchical supervision unit is concatenated with the output histogram Inter_H of the inter-channel granularity-level supervision unit to obtain the final output feature $H_{DDHTS-Net}$ of DDHTS-Net, denoted Equation (18):

(18) $$H_{DDHTS-Net}=[Inter\_H,Intra\_H]$$

Algorithm 1 first lists the selected feature extraction layers for different backbone networks, namely [6,10,12,14] for AlexNet, [9,16,23,30] for VGG16, and [9,18,27,36] for VGG19. Then, for each input image, the algorithm generates three multi-band contrast descriptors and constructs the four input channels consisting of the original image and the three contrast descriptor maps. After extracting and normalizing deep feature maps from the selected backbone, Step 7 obtains the intra-channel hierarchical supervision feature through IntraHSU, and Step 8 obtains the inter-channel granularity-level supervision feature through InterGSU. Finally, Step 9 concatenates the two supervision features to form the final DDHTS-Net representation, which is subsequently used for SVM-based classification.

Algorithm 1 **Pseudocode of the proposed DDHTS-Net with IntraHSU and InterGSU.**

**Input:** X={I_m}_{m=1}^M, labels Y, backbone Phi_B, B in {AlexNet, VGG16, VGG19}, selected layers L_B, and parameter C.
**Output:** Final feature set H_DDHTS-Net and classification results.
1: Set L_B = [6,10,12,14] for AlexNet; [9,16,23,30] for VGG16;  and [9,18,27,36] for VGG19.
2: for each image I_m in X do
3:  Generate multi-band contrast descriptors {D_1,D_2,D_3}; set Q={I_m,D_1,D_2,D_3}.
4:  for each channel q in Q do
5:   Extract and normalize deep feature maps F_q from Phi_B at layers L_B.
6:  end for
7:  H_Intra^m <- IntraHSU(F_I, F_D1, F_D2, F_D3).
8:  H_Inter^m <- InterGSU(F_I, F_D1, F_D2, F_D3).
9:  H_DDHTS-Net^m <- [H_Inter^m, H_Intra^m].
10: end for
11: Train/test SVM using H_DDHTS-Net and report mean +- std over 50 trials.

## 3 Experimental evaluation

To validate the effectiveness of the proposed DDHTS-Net, this section conducts extensive classification performance evaluations on two plant texture datasets - including a plant disease dataset (PlantVillage Dataset (Hughes and Salathé, 2015)) and a plant leaf classification dataset (Swedish Leaf Dataset (Söderkvist, 2001)) - as well as on two publicly available benchmark texture datasets: UMD (Xu et al., 2009) and UIUC database (Lazebnik et al., 2005). **Table 1** presents the detailed information of the datasets.

**Table 1:** Summary of databases.

Database	Total images	Classes	Image size	Experiment evaluation
PlantVillage Dataset	543035	38	unfixed	Classification and recognition of different plant diseases
Swedish Leaf Dataset	1140	15	unfixed	Plant leaf classification and recognition
UIUC	1000	25	640×480	Texture classification under uncontrolled conditions
UMD	1000	25	1280×960	Texture classification under uncontrolled conditions

All experiments were conducted on a workstation configured with dual Intel Xeon Gold 6226R processors (32 cores and 64 threads). Notably, no GPU acceleration was employed throughout the study. The system was equipped with 256 GB of DDR4 RAM and operated under Ubuntu 20.04.6 LTS. The proposed method was implemented in MATLAB R2024b using the MatConvNet framework. A Support Vector Machine (SVM) was adopted as the classifier for all evaluations. In the experiment, we randomly selected 80% of the samples from each category for training and used the remaining 20% for testing, and the final results were obtained by averaging the outcomes over 50 independent randomized trials.

### 3.1 Experiment results on PlantVillage dataset

**Figure 6:**
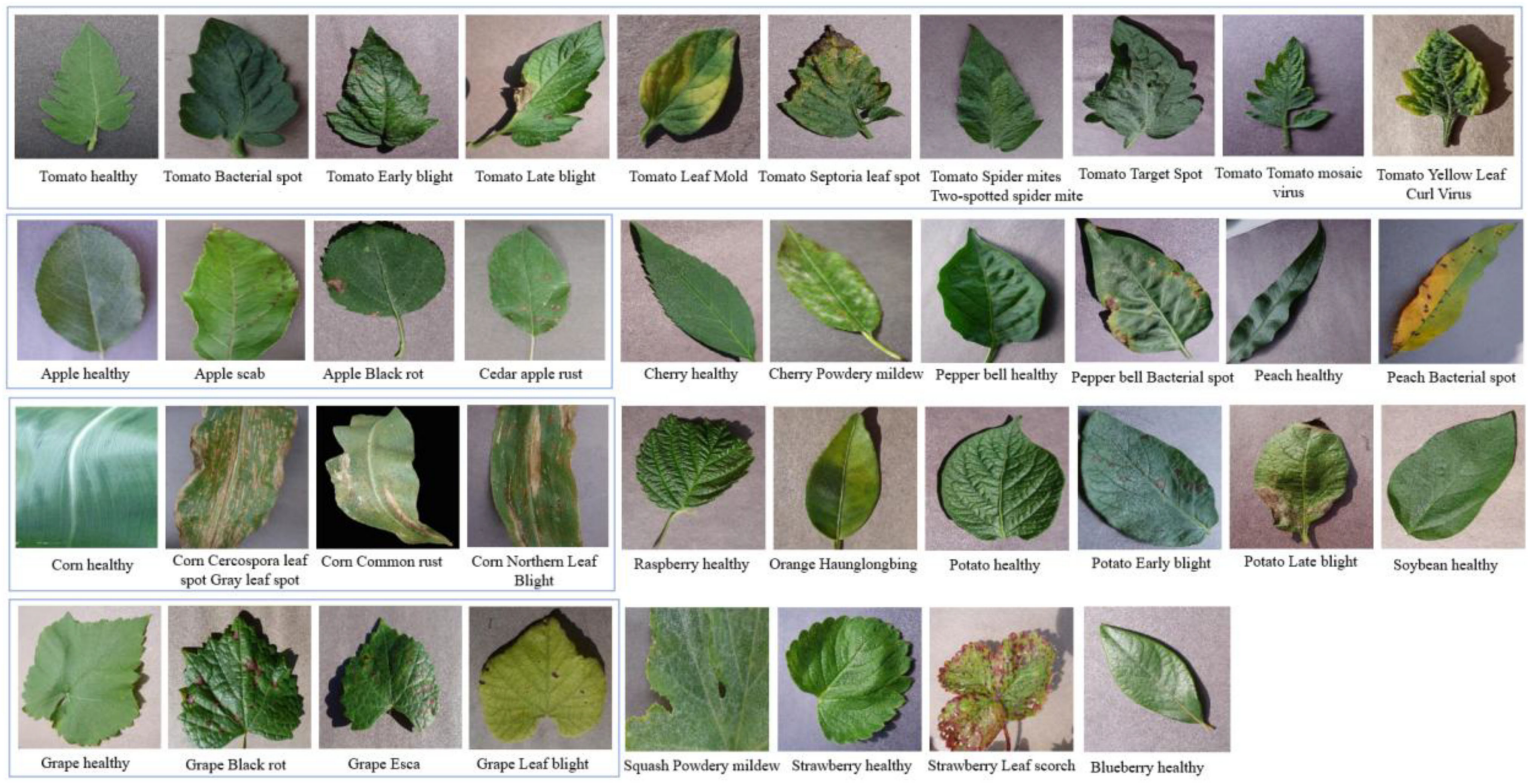
The 38 leaf sample examples from the PlantVillage dataset.

The PlantVillage dataset is a large-scale benchmark specifically curated for plant disease identification and crop health status classification. It is intended to advance crop health monitoring by harnessing machine learning and deep learning techniques. The dataset comprises over 50,000 high-resolution color images, covering 14 crop species (e.g., apple, tomato, grape, corn) and 26 commonly observed plant diseases. Owing to its scale and diversity, the PlantVillage dataset has become a vital resource in the domains of plant pathology and computational agriculture. **Figure 6** presents sample images from the 38 categories of the PlantVillage dataset. In the experiments presented in this section, we evaluated the classification performance of the proposed DDHTS-Net on the complete PlantVillage dataset, encompassing 38 categories. Furthermore, we validated the effectiveness of DDHTS-Net when applied individually to the disease classification tasks of tomato, apple, corn, and grape. These specific disease classification tasks for individual plant species are highlighted in blue boxed areas in **Figure 6**.

**Figure 7:**
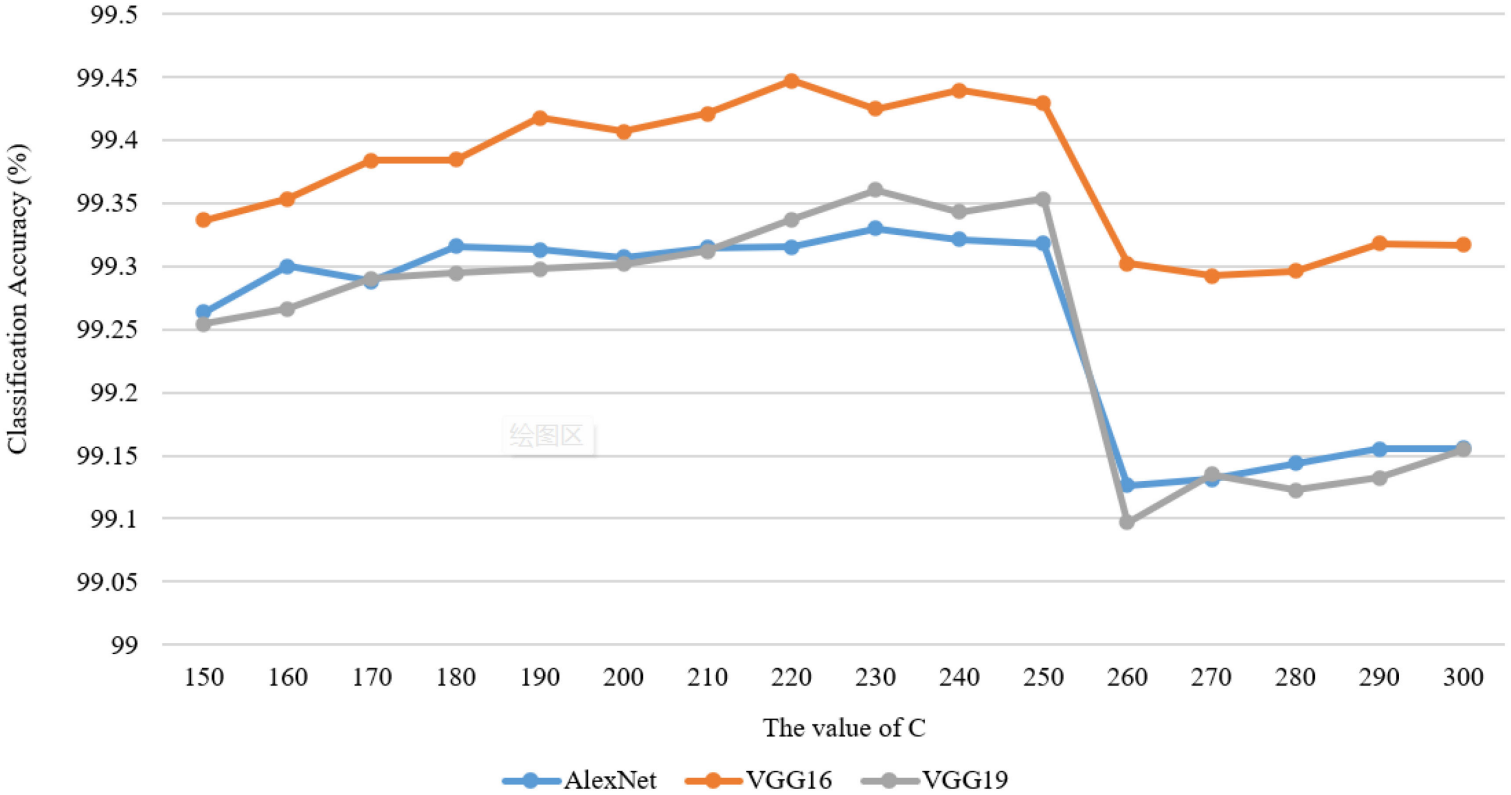
Classification performance of the DDHTS-Net across different backbone networks (AlexNet, VGG16, and VGG19) under varying values of parameter C.

As illustrated in Section 2.2, the number of preserved channels C is a crucial parameter for the normalized deep feature maps. **Figure 7** depicts the classification performance of DDHTS-Net, based on the AlexNet, VGG16, and VGG19 backbone, under varying values of C. It can be observed that the network attains optimal performance when C is appropriately selected. From the **Figure 7**, it can be observed that as the value of C increases, the performance of the DDHTS-Net with different backbone networks also improves gradually. The performance plateaus when C is in the range of 230 to 250. Beyond this range, any further increase in the C value results in a significant degradation of network performance. These experimental findings suggest that setting the C value within the 230 to 250 interval is more favorable for optimal network performance. In this study, we have set C to 250.

**Figure 8:**
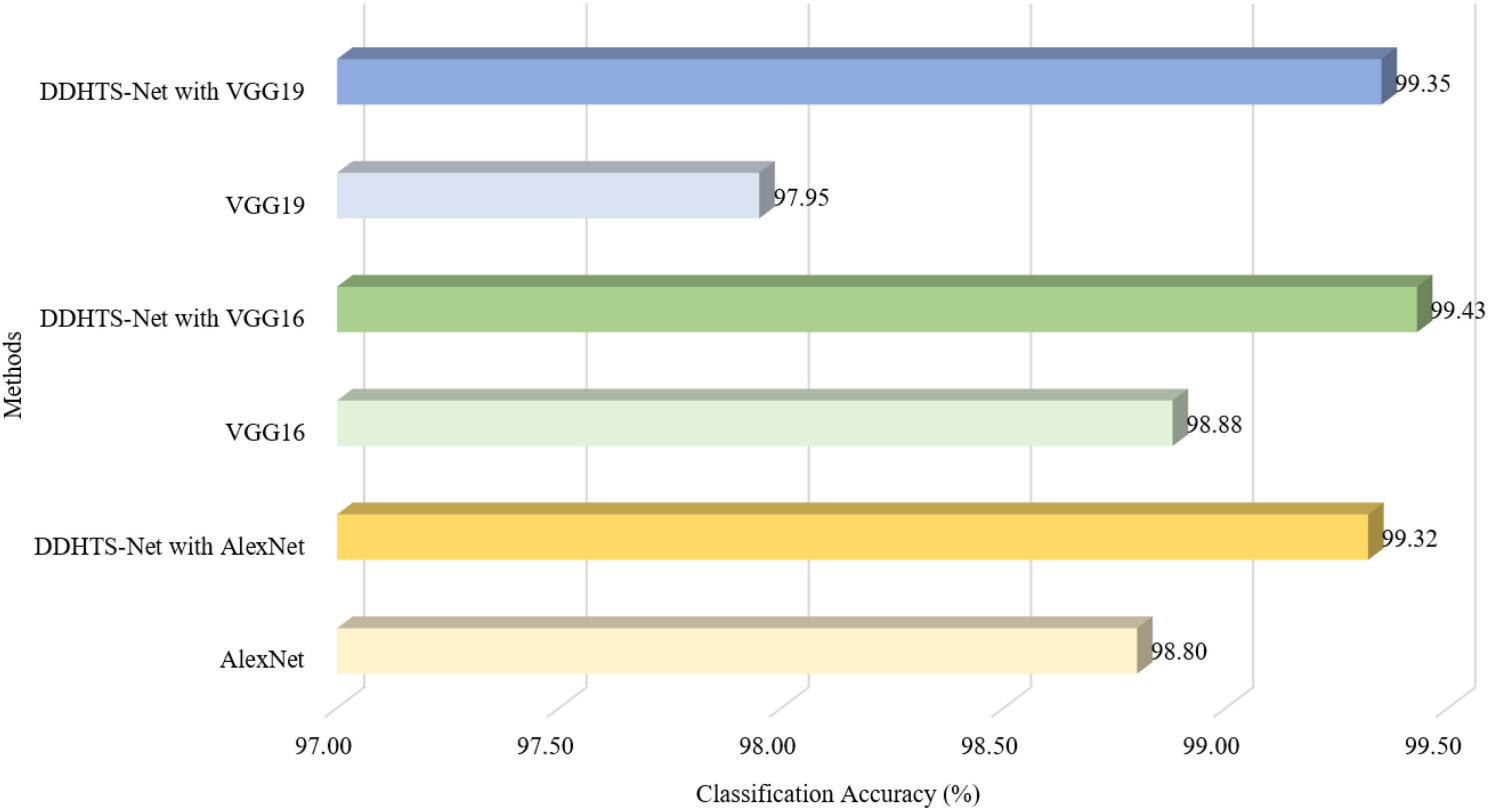
Comparison of the classification performance of the proposed DDHTS-Net with the original backbone networks on the PlantVillage dataset.

To further validate the effectiveness of the proposed DDHTS-Net, **Figure 8** presents the classification results of DDHTS-Net and the original backbone networks on the 38 leaf disease categories in the PlantVillage dataset. As observed in the figure, the classification accuracy of DDHTS-Net is significantly higher than that of its corresponding backbone networks. In particular, DDHTS-Net with AlexNet achieves a 1.40% improvement over the original AlexNet. These results indicate that, compared to the original network frameworks, the proposed DDHTS-Net is better suited to capturing the fine-grained texture details characterized by complex local patterns and spatial structures. Consequently, it effectively addresses the challenges posed by substantial intra-class variability and limited inter-class discriminability inherent in plant leaf disease classification tasks. This superior performance can be primarily attributed to the texture-focused advantages of the multi-band contrast descriptor, as well as the carefully designed intra-channel hierarchical supervision unit and inter-channel granularity-level supervision unit, which collectively enable DDHTS-Net to capture and model complex texture attributes across multiple domains and hierarchical levels.

To validate the effectiveness of the proposed IntraHSU, InterGSU, and the overall DDHTS-Net framework with different backbone networks, **Table 2** presents a comparative analysis of their performance on the 38 classes of samples from the PlantVillage dataset. As shown in **Table 2**, regardless of the backbone network employed, the DDHTS-Net consistently achieves higher classification accuracy across various plant leaf disease categories compared to both IntraHSU and InterGSU. In particular, for the overall classification accuracy across the 38 categories, DDHTS-Net outperforms IntraHSU by 0.34% and slightly surpasses InterGSU when using AlexNet as the backbone network. Similar trends can be observed in the experimental data for the VGG16 and VGG19 backbone networks. Moreover, a comparison of the classification results between IntraHSU and InterGSU reveals that InterGSU consistently outperforms IntraHSU in all categories. For instance, InterGSU with AlexNet, VGG16, and VGG19 achieves overall classification accuracies on the 38 classes of the PlantVillage dataset that are 0.26%, 0.19%, and 0.32% higher, respectively, compared to IntraHSU with the same backbone networks.

**Table 2:** Comparison results (%) of the proposed IntraHSU, InterGSU, and the overall DDHTS-Net framework.

Category	AlexNet			VGG16			VGG19		
	IntraHSU	InterGSU	DDHTS-Net	IntraHSU	InterGSU	DDHTS-Net	IntraHSU	InterGSU	DDHTS-Net
1	98.05	99.16	99.16	98.70	99.16	99.43	98.67	98.84	99.13
2	99.29	99.58	99.61	100.00	99.79	99.87	99.63	99.60	99.94
3	99.85	99.05	99.31	99.75	99.64	99.89	99.82	99.31	100.00
4	99.42	99.55	99.57	99.43	99.72	99.65	99.53	99.68	99.79
5	99.73	99.89	99.93	99.90	99.84	99.90	99.83	99.85	99.87
6	99.25	99.45	99.36	99.56	99.62	99.62	99.40	99.52	99.54
7	99.43	99.70	99.70	99.71	99.86	99.90	99.49	99.77	99.71
8	89.71	90.29	91.39	91.16	90.94	93.02	88.20	90.69	91.27
9	99.82	99.77	99.76	99.83	99.90	99.92	99.83	99.86	99.92
10	99.97	99.93	99.97	100.00	100.00	100.00	100.00	100.00	100.00
11	95.95	95.59	96.05	95.92	95.94	96.88	94.99	96.06	96.38
12	99.07	99.31	99.40	98.97	99.58	99.56	98.70	99.12	99.24
13	99.39	99.53	99.60	99.09	99.60	99.82	98.84	99.32	99.54
14	99.98	100.00	100.00	100.00	100.00	100.00	100.00	100.00	100.00
15	99.90	99.93	99.95	100.00	99.97	100.00	99.96	100.00	100.00
16	99.95	99.95	99.97	99.96	99.97	99.97	99.96	99.96	99.96
17	99.60	99.67	99.73	99.80	99.86	99.82	99.58	99.75	99.75
18	98.97	99.19	99.31	98.53	98.53	98.64	99.19	98.89	99.25
19	98.98	99.30	99.06	99.10	99.24	99.38	98.69	99.47	99.45
20	99.44	99.68	99.69	99.59	99.74	99.83	99.57	99.78	99.72
21	99.87	99.83	100.00	99.78	99.74	99.84	99.38	99.79	99.79
22	91.20	92.73	93.33	93.07	91.53	93.13	90.53	93.27	94.20
23	97.16	97.92	97.83	98.44	98.22	98.55	97.42	98.54	98.70
24	99.16	99.51	99.54	99.86	99.89	99.92	99.95	99.54	99.81
25	99.78	99.85	99.85	99.84	99.90	99.89	99.85	99.84	99.85
26	100.00	99.96	99.98	100.00	100.00	100.00	99.99	100.00	100.00
27	99.67	99.56	99.78	99.78	99.78	99.78	99.63	99.82	99.82
28	99.69	99.80	99.78	99.76	99.92	99.89	99.55	99.75	99.84
29	99.26	99.42	99.52	99.29	99.47	99.62	99.20	99.50	99.47
30	93.95	96.58	96.76	93.66	95.51	96.64	92.25	95.08	95.67
31	99.75	99.81	99.82	99.74	99.72	99.76	99.77	99.66	99.84
32	97.19	98.12	98.32	96.73	97.92	98.14	96.33	97.46	97.95
33	97.87	98.26	98.75	98.00	98.75	99.19	97.28	98.11	98.73
34	98.66	99.21	99.23	98.71	99.11	99.24	98.66	98.75	99.06
35	97.42	98.27	98.52	97.87	98.41	98.79	97.74	98.67	98.96
36	95.81	96.89	97.30	96.61	96.78	97.62	96.69	97.25	97.94
37	99.49	99.78	99.84	99.27	99.84	99.73	99.19	99.30	99.97
38	99.67	99.82	99.83	99.73	99.84	99.86	99.63	99.80	99.82
Total	98.98	99.24	99.32	99.09	99.28	99.43	98.90	99.22	99.35

Furthermore, a comparison of the classification results of DDHTS-Net based on different backbone networks in **Table 2** reveals that the DDHTS-Net with VGG16 as the backbone network achieves the best performance, followed by the DDHTS-Net with VGG19, which slightly outperforms the AlexNet-based DDHTS-Net. These observations suggest that the inter-channel expert information exchange offers a richer source of discriminative texture information than the intra-channel hierarchical expert information exchange. The proposed difference-based inter-channel binary supervision mechanism effectively leverages expert knowledge at different granularity levels to deeply mine the discriminative detail information embedded in the corresponding layers of the original image, thereby providing robust feature support for plant disease classification.

It is worth emphasizing that IntraHSU and InterGSU address two complementary and orthogonal dimensions of texture representation, rather than redundant or interchangeable supervision strategies. Specifically, IntraHSU focuses on hierarchical dependencies within a single input channel, where feature maps at different convolutional depths are treated as hierarchical experts. It captures the progressive evolution from low-level texture primitives (edges, micro-patterns) to high-level abstract representations, thereby modeling cross-layer semantic hierarchy relationships. In contrast, InterGSU focuses on cross-frequency and cross-granularity relationships across different channels, where the original image channel and multi-band transformed channels are compared at the same abstraction level. This mechanism explicitly captures complementary information across spatial frequency bands (e.g., high-frequency detail vs. low-frequency structure), thereby modeling cross-frequency complementarity. Therefore, using only one of these mechanisms captures only a single aspect of texture structure: either hierarchical abstraction (IntraHSU) or frequency-domain complementarity (InterGSU). In contrast, DDHTS-Net jointly exploits both dimensions, enabling a more complete and discriminative representation of texture information. Furthermore, as demonstrated in **Table 2**, the proposed DDHTS-Net consistently outperforms both IntraHSU and InterGSU across all backbone networks. This improvement is not merely incremental, but structurally consistent across 38 categories and three backbone architectures (AlexNet, VGG16, and VGG19), confirming that the two mechanisms are not redundant but mutually reinforcing. In summary, the observed performance gain empirically validates the necessity of combining intra-channel and inter-channel supervision, as they capture complementary properties of texture from different representational perspectives.

To further validate the effectiveness of the proposed DDHTS-Net, the model was employed to perform disease classification tasks across unified plant categories. Specifically, independent evaluations were conducted on apple, corn, grape, and tomato disease classification tasks. Apple, corn and grape datasets each comprise one category of healthy leaves and three categories of diseased leaves. In contrast, the tomato dataset consists of nine disease categories and one category of healthy leaves.

**Figure 9:**
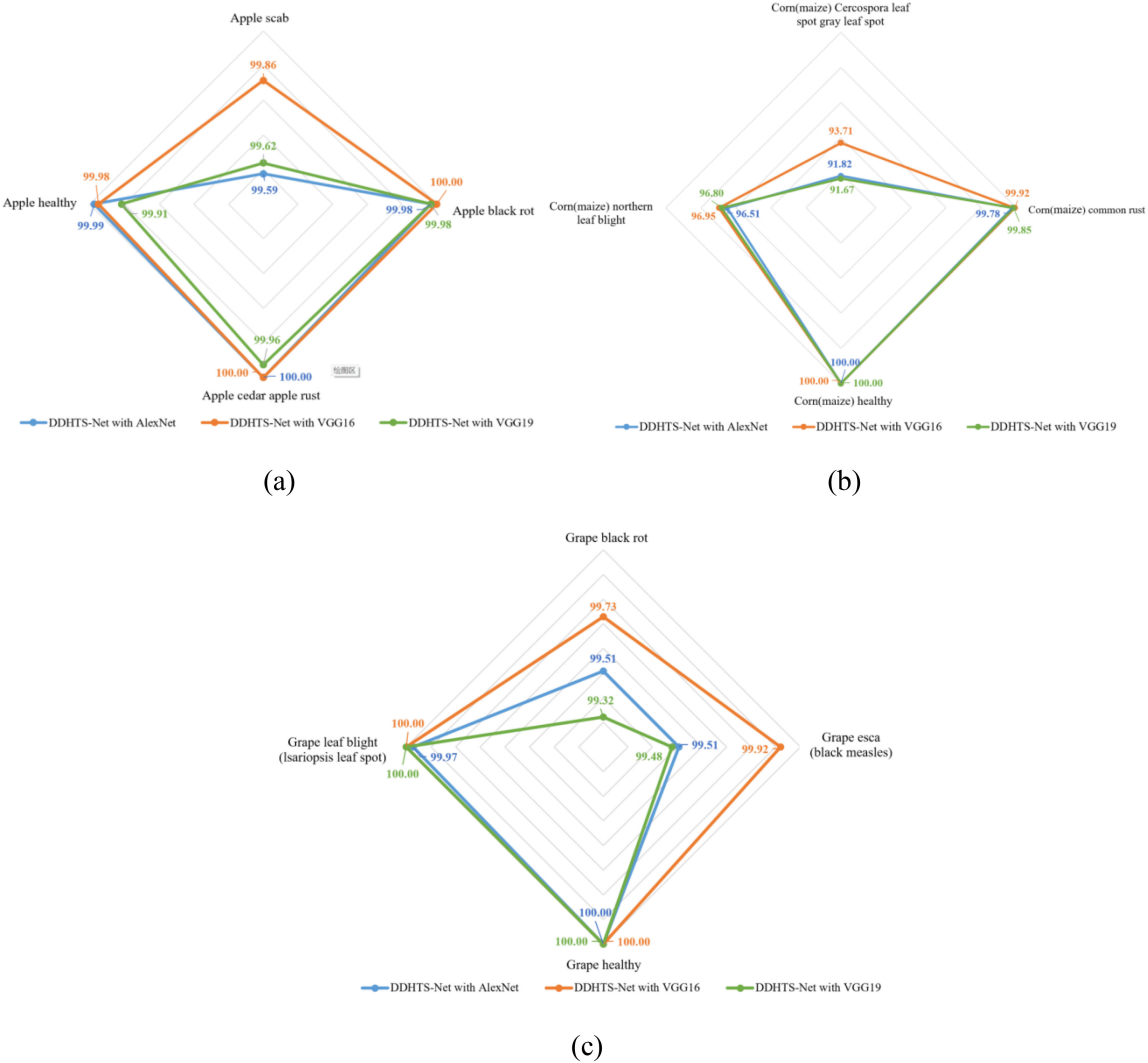
Classification accuracy (%) of DDHTS-Net with different backbone networks for various disease categories in the **(A)** Apple, **(B)** Corn, and **(C)** Grape disease classification tasks.

**Figure 9** presents the classification results of the proposed DDHTS-Net for different disease categories in the apple, corn, and grape disease classification tasks using different backbone networks. As shown in **Figure 9**, DDHTS-Net with VGG16 consistently outperforms the other two networks in classifying leaf diseases of apple, corn, and grape. Specifically, as depicted in **Figure 9A**, the classification performance of DDHTS-Net for the apple leaf disease task is illustrated. For Apple scab, DDHTS-Net with VGG16 achieves the highest classification accuracy, exceeding that of DDHTS-Net with AlexNet by 0.24% and surpassing DDHTS-Net with VGG19 by 0.27%. In contrast, for Apple black rot, Apple cedar apple rust, and Apple healthy leaves, the classification accuracies of DDHTS-Net with all three backbone networks are consistently close to 100%. Overall, DDHTS-Net with VGG16 exhibits superior and more stable classification performance in the apple leaf disease classification task. **Figure 9B** illustrates the classification performance of DDHTS-Net for the corn leaf disease task. Notably, for the most challenging category, Corn (maize) Cercospora leaf spot gray leaf spot, DDHTS-Net with VGG16 attains the highest classification accuracy (93.71%), surpassing that of DDHTS-Net with VGG19 by 1.89% and DDHTS-Net with AlexNet by 2.04%. For the remaining three disease categories, the performance differences among the three networks are negligible. Overall, DDHTS-Net with VGG16 demonstrates superior performance in the corn leaf disease classification task, particularly excelling in the Corn (maize) common rust and Corn (maize) healthy categories, while maintaining relatively stable performance across the different disease classes. **Figure 9C** presents the classification results of DDHTS-Net for the grape leaf disease task. From a comprehensive perspective, DDHTS-Net with VGG16 demonstrates superior overall performance in this task, particularly excelling in the more challenging categories of Grape black rot and Grape esca (black measles), where it clearly outperforms the other two networks. For the Grape healthy and Grape leaf blight (lsariopsis leaf spot) categories, all three networks exhibit exceptionally high classification accuracies.

**Figure 10:**
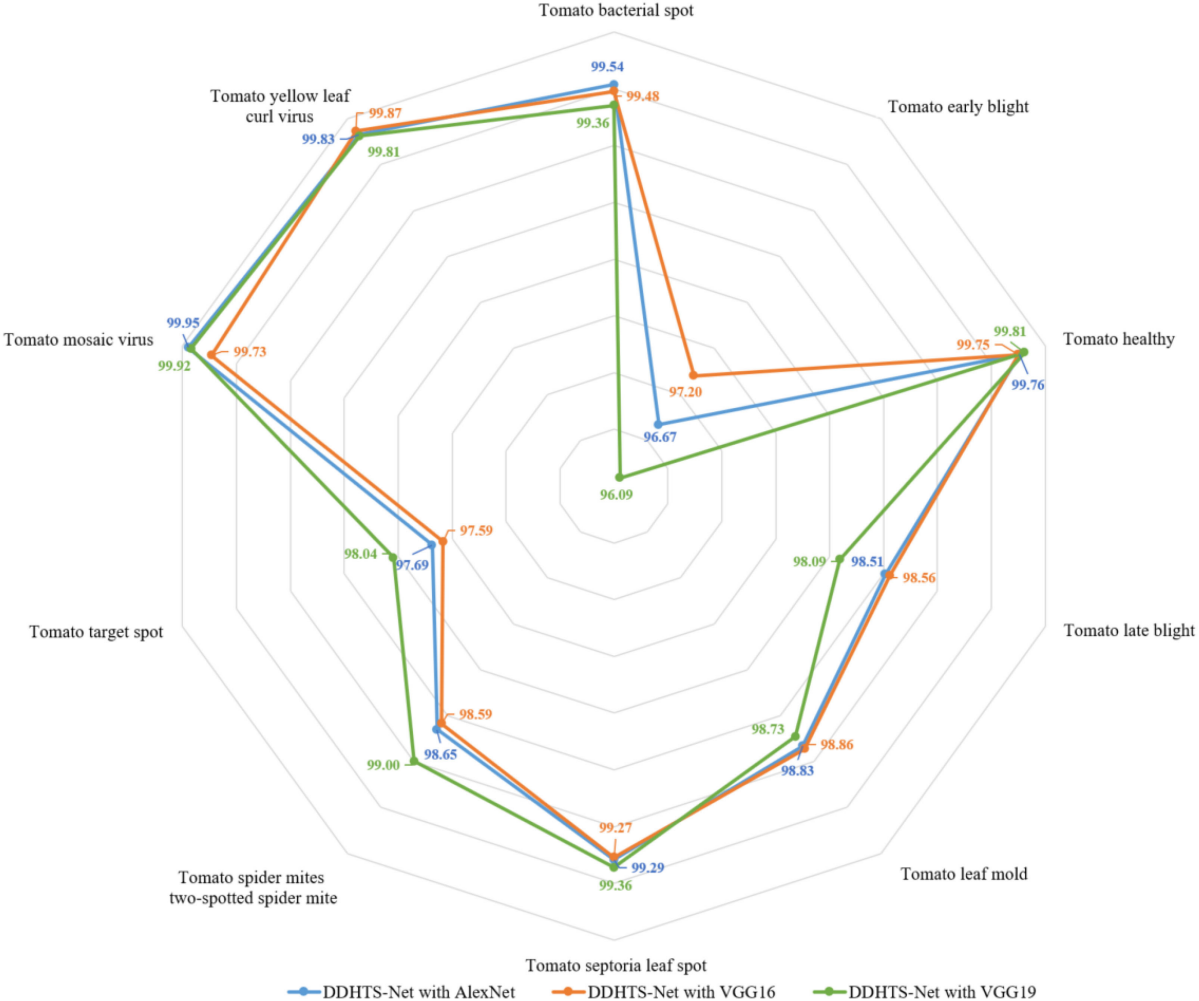
Classification accuracy (%) of DDHTS-Net with different backbone networks for various disease categories in the Tomato disease classification task.

**Figure 11:**
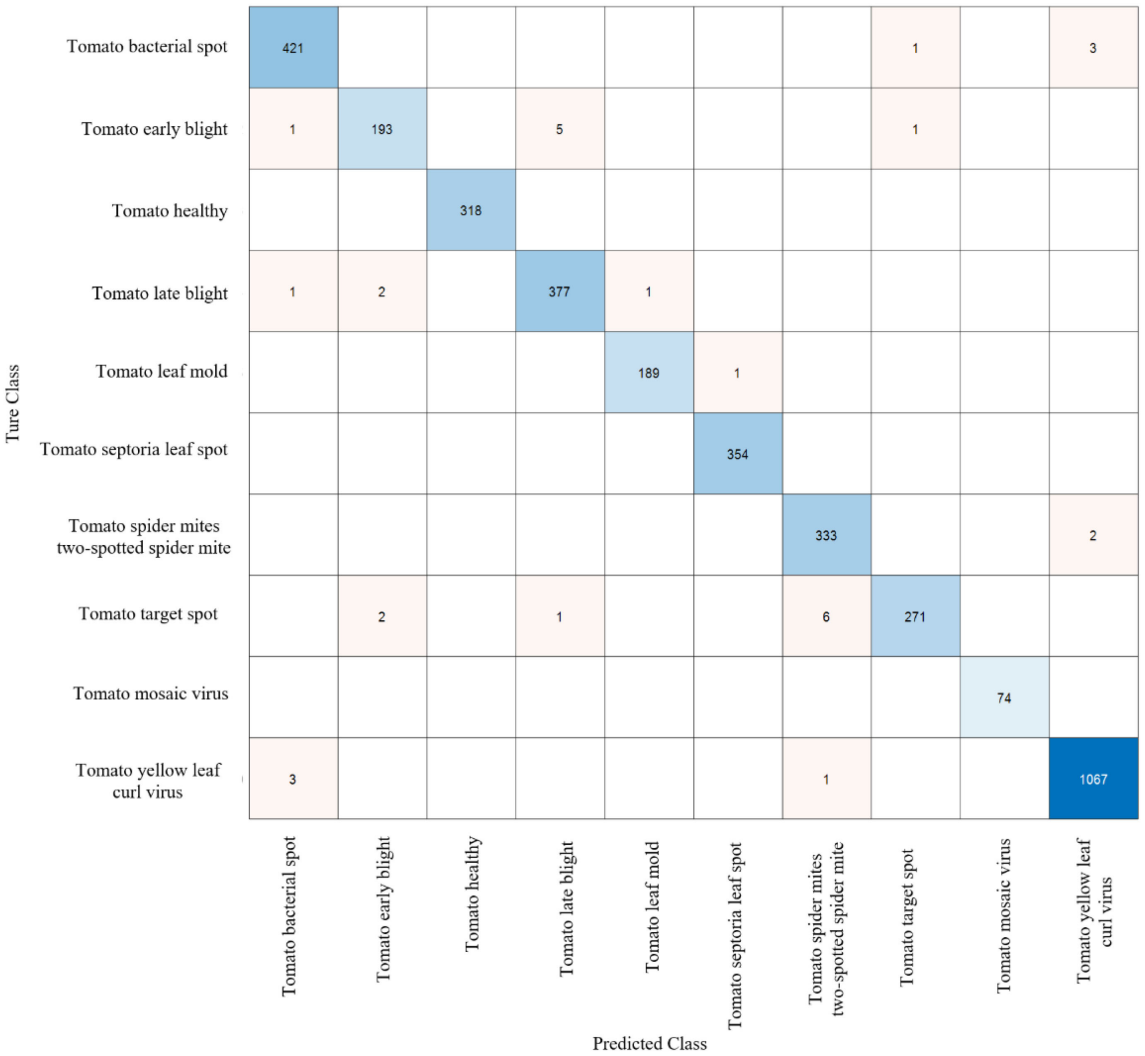
Confusion matrix of DDHTS-Net with AlexNet for the tomato disease classification task.

**Figure 12:**
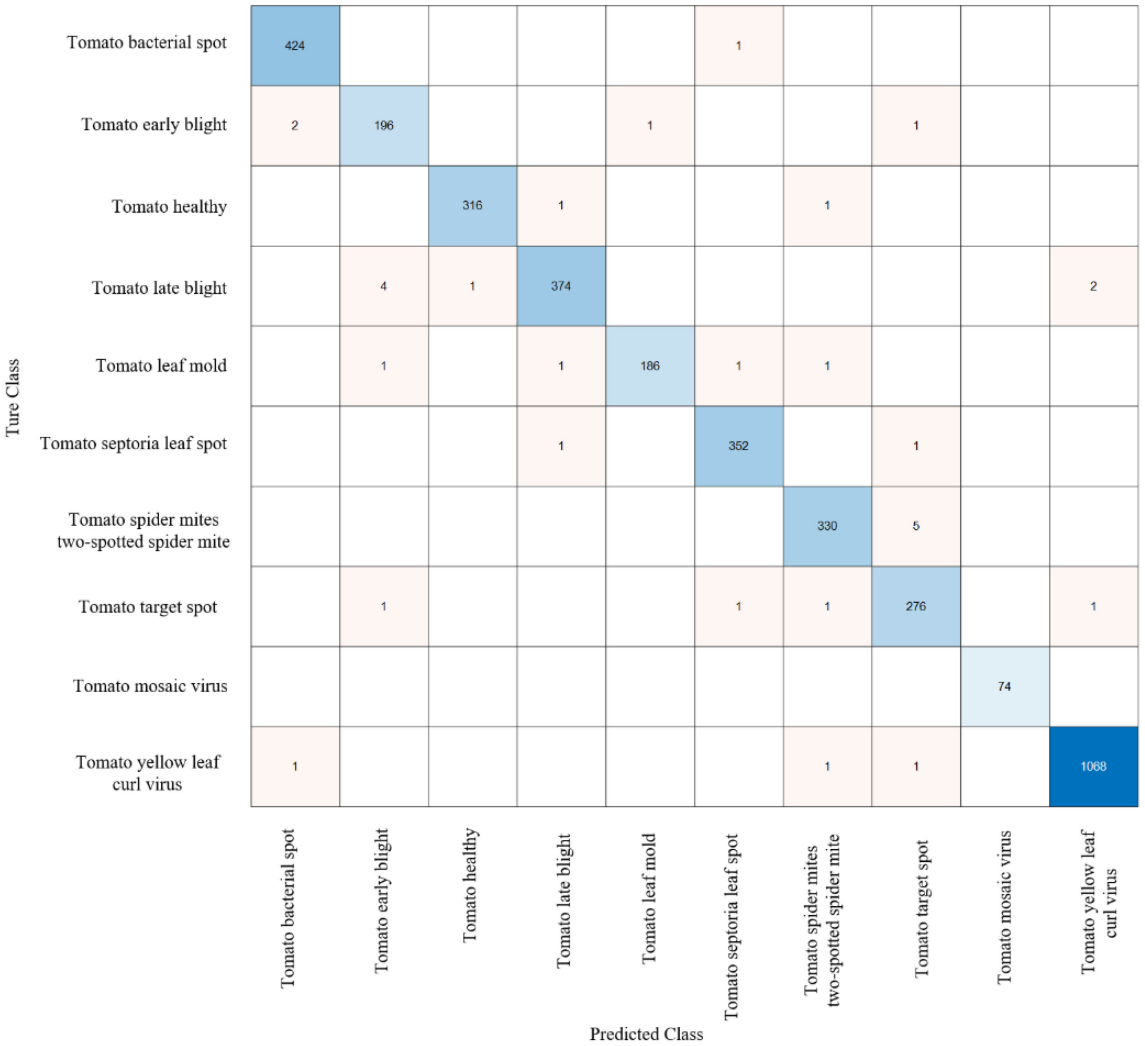
Confusion matrix of DDHTS-Net with VGG16 for the tomato disease classification task.

**Figure 13:**
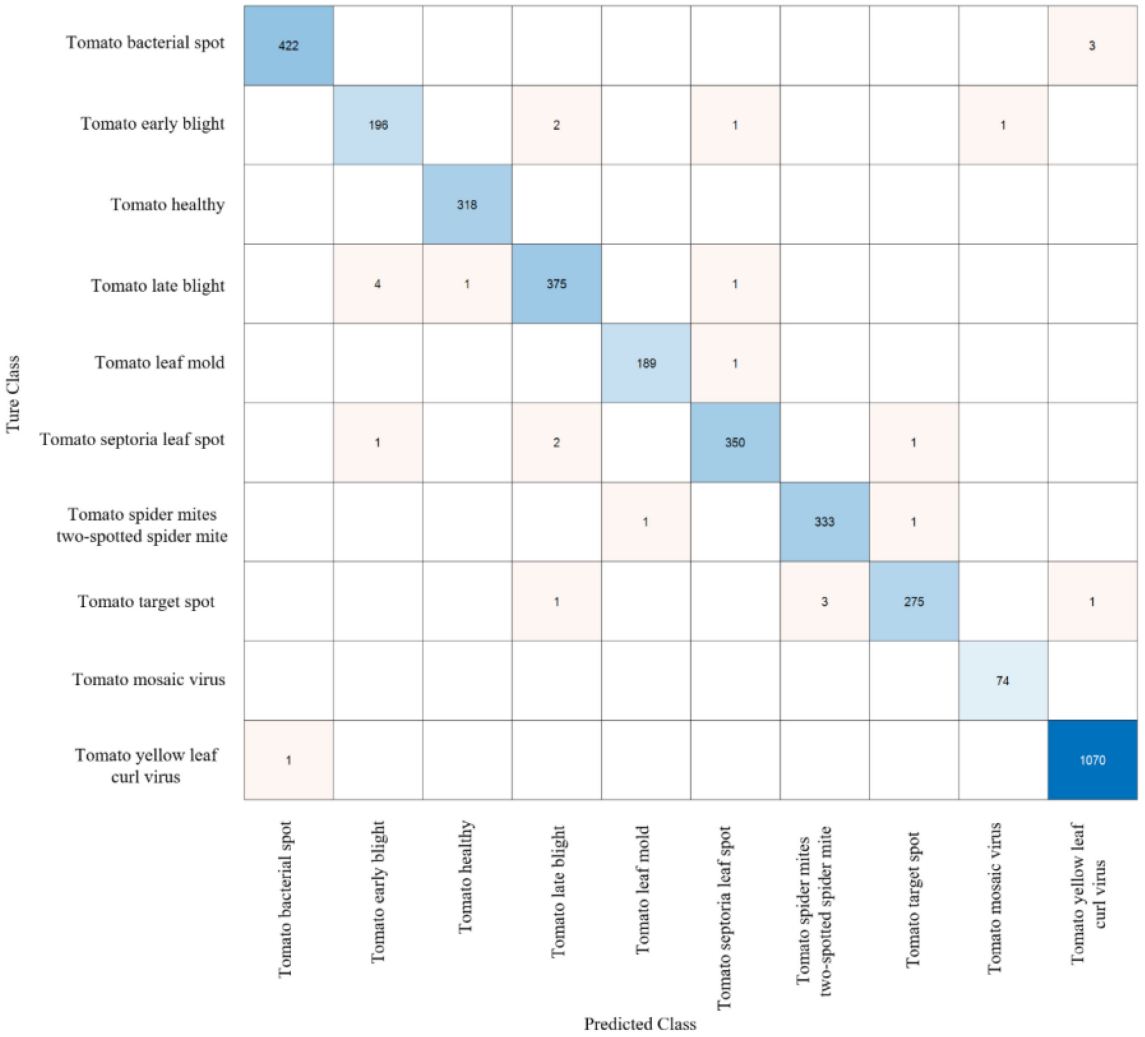
Confusion matrix of DDHTS-Net with VGG19 for the tomato disease classification task.

**Figure 10** illustrates the classification performance of DDHTS-Net with different backbone networks on the tomato leaf disease classification task, which includes nine disease groups and one healthy group. It is evident from **Figure 10** that all three networks exhibit consistently high classification accuracy for the healthy tomato leaf category, thereby demonstrating the efficacy of the proposed DDHTS-Net in distinguishing healthy leaves from diseased ones. Among the disease categories, tomato early blight and tomato target spot present relatively lower classification accuracy. For tomato early blight, the best performance is achieved by DDHTS-Net with VGG16, reaching an accuracy of 97.20%, which surpasses that of DDHTS-Net with AlexNet by 0.53% and DDHTS-Net with VGG19 by 1.11%. In the case of tomato target spot, DDHTS-Net with VGG19 achieves the highest classification accuracy of 98.04%, exceeding that of DDHTS-Net with AlexNet and DDHTS-Net with VGG16 by 0.35% and 0.45%, respectively. **Figures 11**–**13** depict the confusion matrices of the proposed DDHTS-Net employing AlexNet, VGG16, and VGG19 as backbone networks for the tomato leaf disease classification task. These figures comprehensively illustrate the classification outcomes and highlight the differential performance of DDHTS-Net with various backbone architectures. Notably, the categories tomato spider mites two-spotted spider mite and tomato mosaic virus exhibit a higher propensity for misclassification across the models. However, DDHTS-Net utilizing VGG19 demonstrates an enhanced discriminative capability in distinguishing these two disease categories. Conversely, in the case of the more frequently confused categories, tomato early blight and tomato late blight, DDHTS-Net with VGG16 outperforms the other configurations, indicating superior recognition performance. Overall, DDHTS-Net with VGG16 and VGG19, as deeper backbone networks, generally achieve superior classification performance across most categories, particularly in capturing fine-grained leaf disease texture features. However, DDHTS-Net with AlexNet demonstrates slightly better performance for certain categories, such as tomato bacterial spot and tomato mosaic virus, suggesting that its shallower architecture still offers advantages in extracting specific local features.

**Figure 14:**
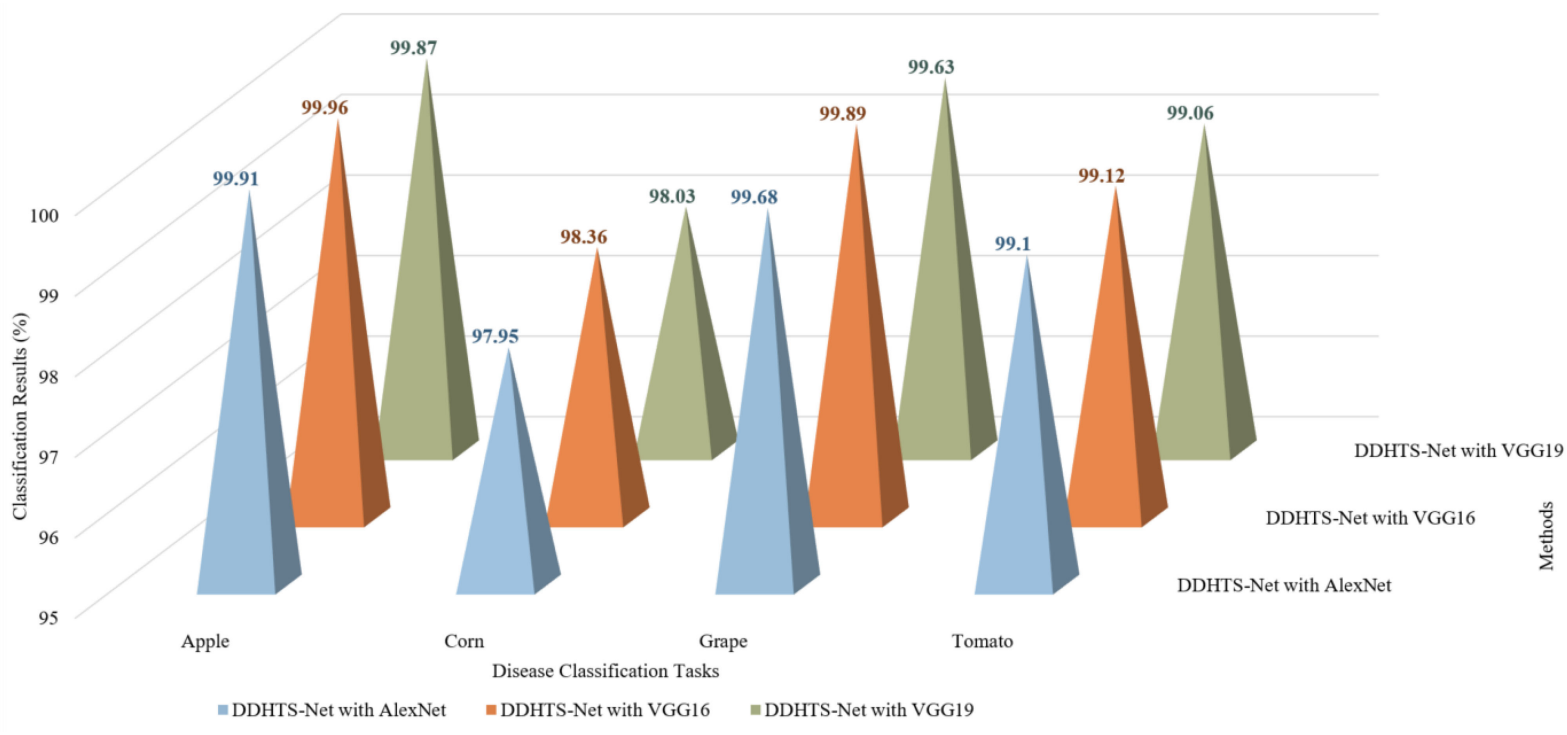
Classification accuracy (%) of DDHTS-Net with different backbone networks for Apple, Corn, Grape, and Tomato disease classification tasks.

**Figure 14** presents the overall classification results of DDHTS-Net with different backbone networks on four plant leaf disease classification tasks. A comprehensive analysis of the experimental results presented in **Figure 14** for four plant leaf disease classification tasks indicates that DDHTS-Net with VGG16 consistently demonstrates superior overall performance. For texture analysis tasks, VGG16, as a 16-layer backbone network, achieves an optimal balance between network depth and feature extraction capacity, enabling the extraction of richer local spatial patterns and contextual texture features. In contrast, AlexNet, with its shallower architecture, may lack the ability to capture fine-grained features in complex texture distributions associated with disease patterns. Although VGG19 is deeper, it can introduce feature redundancy or lead to the loss of fine details in specific tasks, resulting in slightly inferior performance compared to VGG16 in certain disease categories.

**Figure 15:**
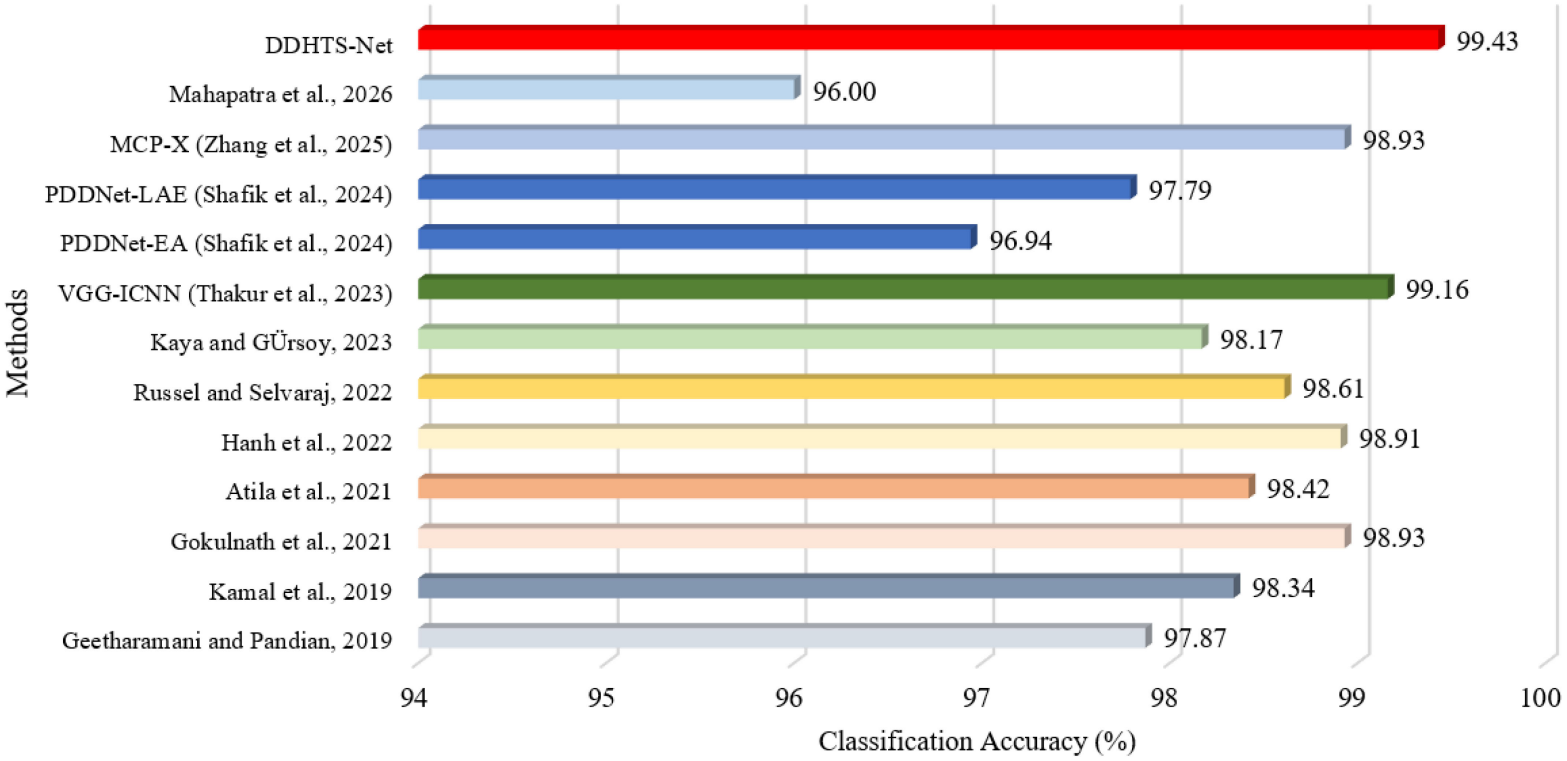
Comparison of the classification accuracy of DDHTS-Net with recent state-of-the-art methods on the whole PlantVillage dataset.

**Figure 15** illustrates the classification accuracy comparison between DDHTS-Net and several advanced methods proposed in recent years on the whole PlantVillage dataset. The compared methods in **Figure 15** include the approaches reported by Geetharamani and Pandian (2019), Kamal et al. (2019), Gokulnath (2021), Atila et al. (2021), Hanh et al. (2022), Russel and Selvaraj (2022), Kaya and Gürsoy (2023), Thakur et al. (2023), Shafik et al. (2024), Mahapatra et al. (2026), and Zhang et al. (2025). The proposed DDHTS-Net achieves the highest classification accuracy of 99.43%, followed by the VGG-ICNN model, which achieves an accuracy of 99.16%, and significantly outperforms all other competing approaches. In particular, DDHTS-Net surpasses the PDDNet-LAE and PDDNet-EA models, proposed in 2024, by 1.64% and 2.49%, respectively. In addition, compared with the recently proposed ultra-lightweight method MCP-X, the proposed DDHS-Net still demonstrates a clear performance advantage. Similar trends are observed when comparing DDHTS-Net with earlier methods, further validating its superior performance. These findings substantiate the effectiveness and superiority of DDHTS-Net in capturing complex leaf texture patterns, underscoring its promising potential for plant disease classification and related texture analysis applications.

Its outstanding performance can be attributed to two key factors. Firstly, DDHTS-Net employs the undecimated wavelet transform (UWT) to construct four spatial frequency channels, including the original image. The high-frequency channels are dedicated to capturing intricate local texture details, whereas the low-frequency channels focus on extracting global structural and contour information. This frequency band partitioning strategy effectively mimics the hierarchical processing of texture information in the human visual system, thereby enhancing the model’s perception of complex texture features and improving its generalization capability across diverse texture classification tasks. Secondly, the intra-channel hierarchical supervision unit leverages the differences between adjacent deep feature maps to establish a binary supervision mechanism. This mechanism enables higher-level “experts” to guide their lower-level counterparts in transmitting discriminative texture information, thereby enhancing the fine-grained differentiation of local features and facilitating the integration of deep representations with shallow details. This process aids in uncovering latent and critical texture attributes. Furthermore, the inter-channel granularity supervision unit facilitates collaboration among experts at different granularity levels, enabling them to jointly supervise and guide the texture information embedded in the original image channel. This cross-channel and cross-level cooperative encoding strategy further promotes the effective modeling and perception of complex cross-domain texture attributes, thereby improving the model’s adaptability to varying texture distributions.

In summary, DDHTS-Net exhibits notable technical advantages by seamlessly integrating multi-band texture information partitioning with hierarchical supervision mechanisms. This integration not only enhances the network’s capability to capture intricate texture details but also improves the complementarity of feature representations across different layers and strengthens the adaptability to cross-domain variations. These attributes collectively enable DDHTS-Net to achieve superior performance compared to traditional convolutional networks in a wide range of plant texture analysis tasks.

### 3.2 Experiment results on Swedish leaf dataset

**Figure 16:**
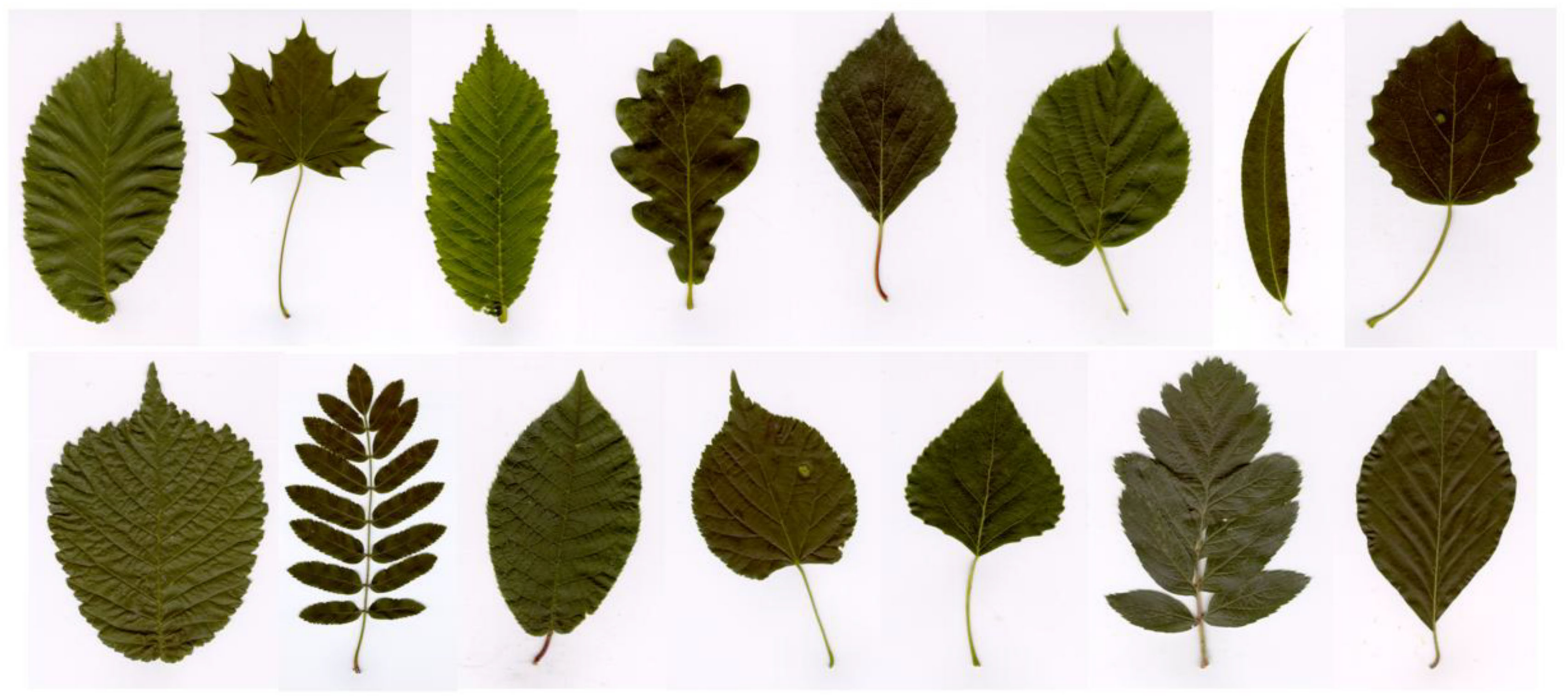
Representative sample images of the Swedish leaf dataset.

To further demonstrate the effectiveness of the proposed DDHTS-Net in plant leaf classification tasks, a series of evaluation experiments were conducted on the Swedish Leaf Dataset. This dataset is specifically designed for leaf classification and is widely used to evaluate image recognition algorithms based on shape and texture features. **Figure 16** presents representative sample images from the Swedish Leaf Dataset.

**Figure 17:**
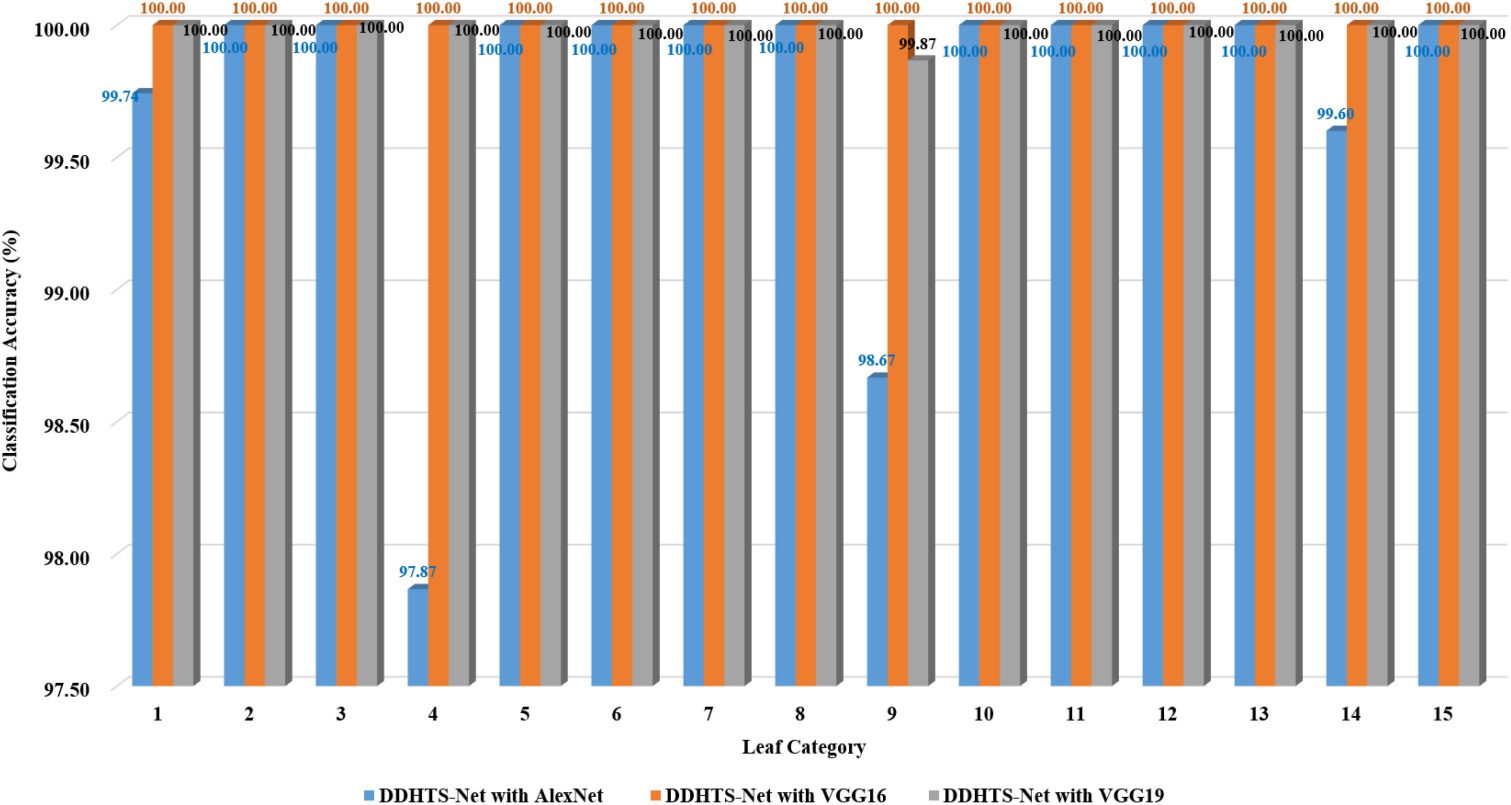
The classification accuracy for each category on the Swedish leaf dataset using the DDHTS-Net framework with different backbone networks.

**Figure 17** illustrates the classification accuracy for each category on the Swedish Leaf Dataset using the DDHTS-Net framework with different backbone networks. As shown in **Figure 17**, DDHTS-Net achieves outstanding classification performance on the Swedish Leaf Dataset, particularly when combined with VGG-based backbone networks. Notably, DDHTS-Net with VGG16 and VGG19 attains 100% classification accuracy in nearly all categories, demonstrating exceptional stability and robustness. Although AlexNet provides a more lightweight architecture, DDHTS-Net with AlexNet exhibits slightly inferior performance. While it achieves 100% accuracy in most categories, noticeable declines are observed in Category 1 (99.74%), Category 4 (97.87%), Category 9 (98.67%), and Category 14 (99.60%) compared to the VGG-based variants. Consequently, VGG16 and VGG19 are recommended as preferred backbone networks for practical deployments, as they offer more consistent and reliable classification performance.

**Table 3** presents a comprehensive comparative analysis of classification performance on the Swedish Leaf Dataset, including the proposed DDHTS-Net with different backbone networks, the corresponding baseline backbones, and several representative state-of-the-art methods. Overall, the results clearly demonstrate that the proposed DDHTS-Net consistently achieves superior performance across all settings. In particular, DDHTS-Net reaches 100.00% accuracy with VGG16 and 99.99% with VGG19, while even the lightweight AlexNet backbone achieves 99.74%, indicating strong robustness and generalization capability across different network capacities. From a methodological perspective, the comparison with the original backbone networks (AlexNet, VGG16, and VGG19) is used to directly verify the effectiveness of the proposed supervision mechanism. The significant performance improvements over standalone CNNs (e.g., VGG16: 95.84%, VGG19: 96.32%, AlexNet: 95.75%) clearly indicate that the proposed IntraHSU and InterGSU modules effectively enhance feature discriminability by introducing cross-layer and cross-frequency supervision. The comparison with recent deep learning-based methods such as Deep-Plant (97.54%), AE-Net (97.90%), and PMG (98.10%) is intended to evaluate the competitiveness of DDHTS-Net against advanced feature learning architectures. Although these methods already achieve strong performance, they still rely primarily on single-stream deep representations, whereas the proposed method explicitly models both hierarchical feature dependencies and frequency-domain complementarity, resulting in consistently higher accuracy. Furthermore, feature fusion-based methods such as MTD + LBP-HF (98.48%) and IMTD + relu5_2 (99.47%) are included to assess the effectiveness of multi-feature integration strategies. While these approaches improve performance by combining heterogeneous descriptors, they lack an explicit cross-channel supervision mechanism, which limits their ability to fully exploit inter-frequency interactions. In contrast, DDHTS-Net integrates multi-band contrast information through a unified supervision framework, leading to more effective feature collaboration. In addition, comparisons with dual-path and multi-branch architectures, including Dual-path CNN (96.28%), S2CL-Leaf Net (99.30%), and S-ResNet (91.70%), are conducted to evaluate the ability of the proposed method in modeling complex texture structures. The results further confirm that explicitly designed hierarchical and cross-frequency supervision is more effective than simply increasing network branching complexity. In summary, **Table 3** not only demonstrates that DDHTS-Net achieves state-of-the-art performance on the Swedish Leaf Dataset, but also validates the effectiveness of each component design. The multi-band contrast descriptor enhances frequency diversity, IntraHSU strengthens cross-layer feature refinement, and InterGSU improves cross-channel complementarity, jointly leading to a more discriminative and robust texture representation framework.

**Table 3:** Comparison results (%) of the proposed DDHTS-Net with state-of-the-art methods reported in the literature.

Method	Classification accuracy (%)
AlexNet (Krizhevsky et al., 2012)	95.75
VGG19 (Simonyan and Zisserman, 2014)	96.32
VGG16 (Simonyan and Zisserman, 2014)	95.84
Deep-Plant (Lee et al., 2017)	97.54
Dual-path CNN (Shah et al., 2017)	96.28
S-ResNet (Wang and Wang, 2019)	91.70
S-Inception (Wang and Wang, 2019)	88.80
PMG (Du et al., 2020)	98.10
AE-Net (Hu et al., 2021)	97.90
MTD + LBP-HF (Yang, 2021)	98.48
S2CL-Leaf Net (Zou et al., 2023)	99.30
IMTD + relu5_2 (Wu et al., 2023)	99.47
DDHTS-Net with AlexNet	99.74
DDHTS-Net with VGG16	100.00
DDHTS-Net with VGG19	99.99

### 3.3 Experiment results on two texture classification databases

**Figure 18:**
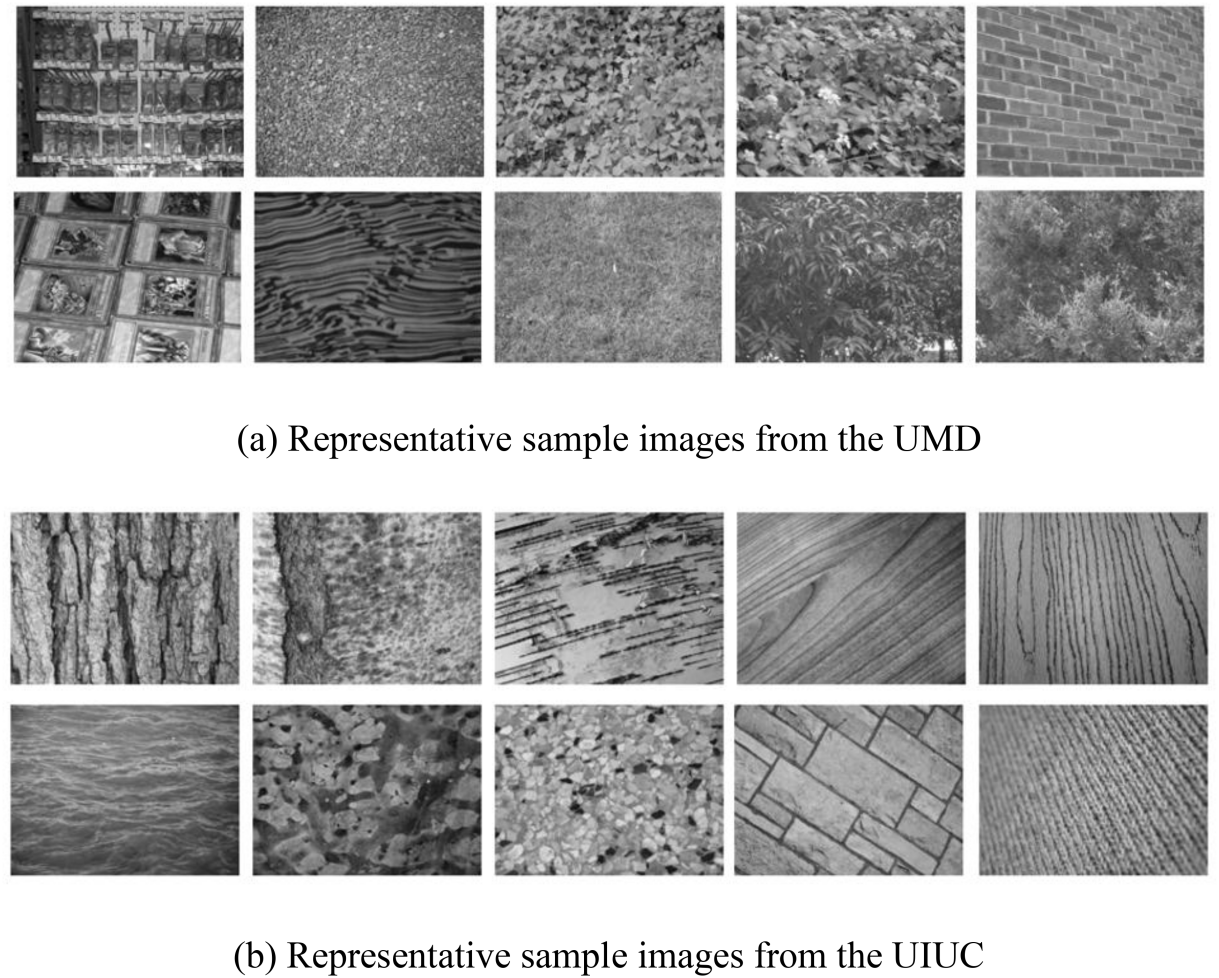
Representative sample images of the two texture classification databases: **(a)** UMD texture database and **(b)** UIUC texture database.

To further assess the effectiveness of the proposed DDHTS-Net on benchmark texture classification tasks, comprehensive evaluation experiments were conducted on three public texture datasets: UMD and UIUC. **Figure 18** presents representative sample images from each dataset.

**Figure 19:**
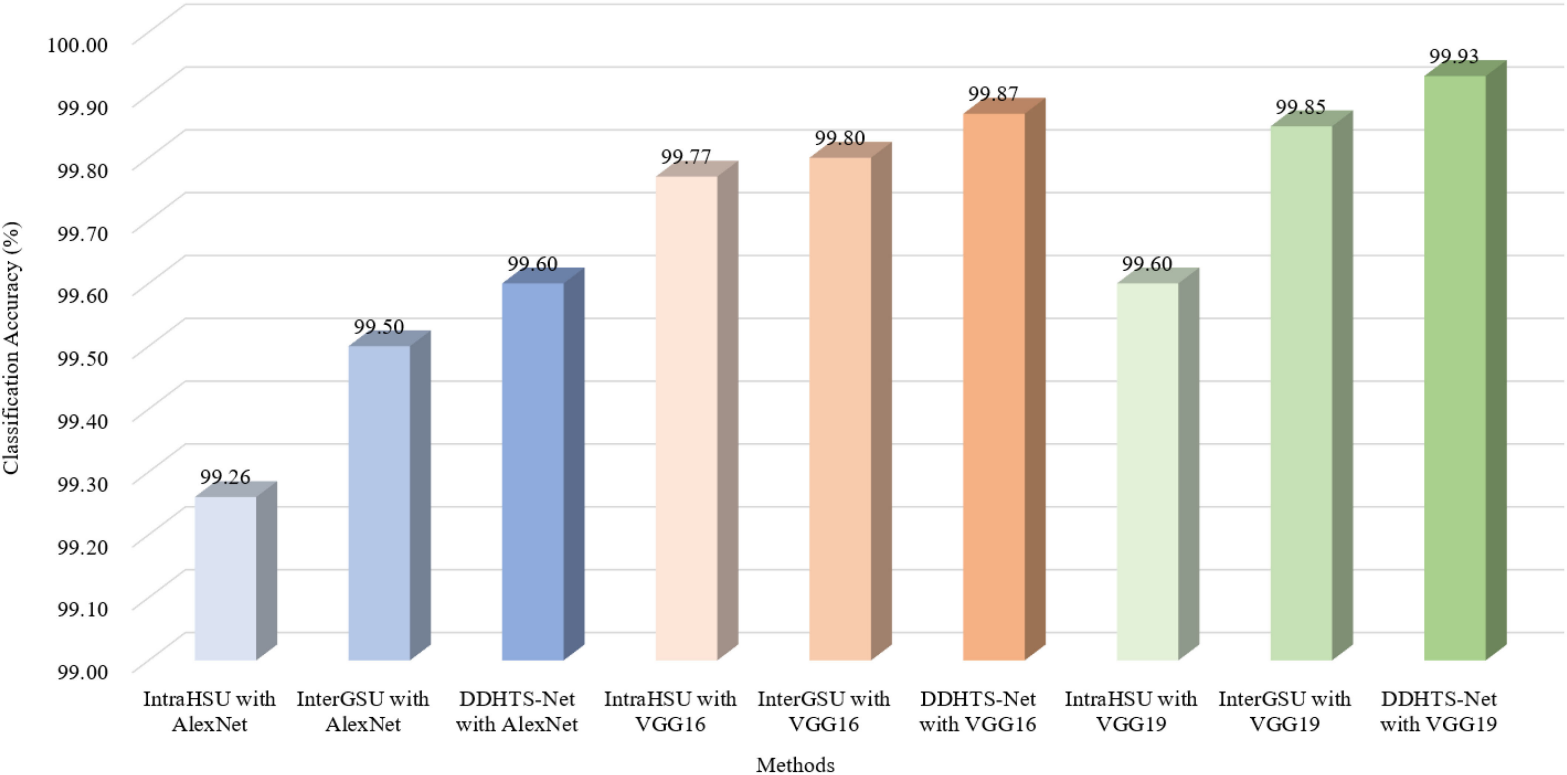
Comparison of classification performance on UMD database using different backbone networks for the proposed IntraHSU, InterGSU, and the overall DDHTS-Net.

To validate the effectiveness of the proposed IntraHSU, InterGSU, and the overall DDHTS-Net framework, **Figure 19** presents a comparison of classification performance based on different backbone networks on UMD database. As illustrated by the experimental results in **Figure 19**, DDHTS-Net consistently achieves higher classification accuracy than both IntraHSU and InterGSU when applied to the same backbone network. This indicates that the effective integration of IntraHSU and InterGSU within the DDHTS-Net architecture facilitates the extraction of complementary information among multi-depth expert feature maps generated by the multi-band contrast descriptor, thereby enhancing the overall recognition capability. Specifically, when employing VGG19 as the backbone, DDHTS-Net improves classification accuracy by 0.33% and 0.08% compared to IntraHSU and InterGSU, respectively. Among the three evaluated backbones, DDHTS-Net with VGG19 achieves the highest performance, outperforming its counterparts based on VGG16 and AlexNet. Moreover, a comparison between IntraHSU and InterGSU reveals that InterGSU exhibits slightly superior performance. This suggests that leveraging expert supervision at multiple granularity levels within the same abstraction layer can more effectively guide feature extraction from corresponding image channels, promoting cross-network interaction and enabling robust modeling of complex cross-domain texture attributes.

As shown in **Table 4**, the average classification accuracy of DDHTS-Net with VGG16 is slightly higher than that of DDHTS-Net with VGG19, and both outperform DDHTS-Net with AlexNet. Notably, DDHTS-Net with VGG16 achieves 100% accuracy in 19 texture categories, whereas DDHTS-Net with AlexNet attains perfect accuracy in only 9 categories. Additionally, texture categories 17 and 25 reach 100% classification accuracy across all three network variants, indicating that these textures possess distinctive features that are easily recognizable. In contrast, category 6 achieves only 92.74% accuracy with DDHTS-Net based on AlexNet, while the accuracy rises above 99.50% when using VGG16 and VGG19, demonstrating that deeper networks are more effective in capturing complex texture patterns. Therefore, VGG16 can be considered the most optimal backbone network for the UIUC texture classification task, as it offers both high accuracy and low variability. Although AlexNet is computationally efficient and lightweight in terms of parameters, it shows clear limitations in fine-grained texture recognition.

**Table 4:** The classification result (%) for each category on UIUC Dataset using the DDHTS-Net framework with different backbone networks.

Texture category	DDHTS-Net with AlexNet	DDHTS-Net with VGG16	DDHTS-Net with VGG19
1	98.25	100.00	99.07
2	98.00	96.50	98.00
3	95.75	99.75	97.25
4	100.00	100.00	97.50
5	99.25	99.50	99.25
6	92.75	99.75	99.50
7	100.00	99.75	98.00
8	96.50	100.00	100.00
9	94.00	100.00	100.00
10	97.25	97.75	100.00
11	99.25	100.00	98.25
12	99.25	97.75	100.00
13	97.50	99.75	98.75
14	97.25	95.00	100.00
15	99.25	100.00	96.75
16	100.00	100.00	99.50
17	100.00	100.00	100.00
18	98.75	98.75	100.00
19	99.00	99.75	98.25
20	99.25	98.00	100.00
21	99.75	100.00	96.25
22	100.00	100.00	100.00
23	97.25	99.50	100.00
24	100.00	100.00	99.50
25	100.00	100.00	100.00
Average	98.33	99.26	99.03

**Table 5** presents a comparative analysis of classification performance between several state-of-the-art methods and the proposed DDHTS-Net on the UMD and UIUC texture datasets. As observed, the classification accuracies on the UIUC dataset are generally lower than those on the UMD dataset across all methods, indicating that the UIUC dataset poses greater challenges due to its more complex texture variations and higher intra-class diversity. For the UMD dataset, DDHTS-Net with VGG19 achieves the highest accuracy of 99.93%, outperforming all other compared methods. Furthermore, under the same backbone architecture (AlexNet), DDHTS-Net with AlexNet surpasses FC-CNN with AlexNet by 3.70%, highlighting the effectiveness of the proposed feature representation framework. Similar trends are observed on the UIUC dataset. Notably, DDHTS-Net with VGG16 achieves the best classification performance of 99.26%, and DDHTS-Net with AlexNet outperforms FC-CNN with AlexNet by a substantial margin of 7.23%. In addition, compared with the DMCE-Net with AlexNet proposed in 2025, the proposed DDHTS-Net still demonstrates a clear performance advantage on both the UIUC and UMD datasets. In summary, the proposed DDHTS-Net consistently outperforms all existing methods on both datasets, with the VGG16- and VGG19-based variants exhibiting exceptional accuracy. Although DDHTS-Net with AlexNet demonstrates slightly lower performance compared to DDHTS-Net with VGG16 and VGG19, it still surpasses all other benchmark methods. This confirms that the proposed framework maintains strong texture recognition capabilities even under lightweight network configurations.

**Table 5:** Comparison results (%) of the proposed DDHTS-Net with state-of-the-art methods on UMD and UIUC database.

Database Method	UMD	UIUC
DeCAF (Cimpoi et al., 2014)	96.40	94.20
FC-CNN with VGGM (Cimpoi et al., 2015)	97.20	94.50
FC-CNN with AlexNet (Cimpoi et al., 2015)	95.90	91.10
DSTNet (Florindo, 2020)	98.50	93.60
VisGraphNet (Florindo et al., 2021)	98.40	98.00
Deep Fractal Interpolation (Florindo and Bruno, 2022)	97.40	96.50
ELMP-Net (Florindo et al., 2024)	99.20	96.30
DMCE-Net with AlexNet (Xu et al., 2025)	99.37	96.94
DDHTS-Net with AlexNet	99.60	98.33
DDHTS-Net with VGG16	99.87	99.26
DDHTS-Net with VGG19	99.93	99.03

The superiority of the proposed DDHTS-Net can be attributed to three key aspects. First, the proposed Undecimated Wavelet Transform (UWT)-based multi-band contrast descriptor explicitly decomposes texture information into multiple spatial frequency and granularity-aware channels. In this formulation, high-frequency channels emphasize fine-grained details such as edges and local variations, while low-frequency channels preserve global structural and contour information. This frequency-aware decomposition enables a more comprehensive and complementary representation of texture patterns across different scales. Second, the intra-channel hierarchical supervision unit (IntraHSU) models cross-layer abstraction relationships by exploiting the differences between adjacent deep feature maps. This mechanism allows higher-level semantic representations to progressively guide lower-level feature learning, thereby facilitating the effective discovery of discriminative low-level texture attributes and improving hierarchical feature consistency within each channel. Third, the inter-channel granularity-level supervision unit (InterGSU) explicitly models cross-frequency interactions by comparing feature representations between the original image channel and multi-frequency transformed channels at the same abstraction level. This design enhances the complementarity among different frequency domains and alleviates the bias introduced by relying on a single representation stream, thereby improving the robustness and discriminability of learned features. Overall, these mechanisms enable DDHTS-Net to go beyond the dependence on backbone networks alone. Instead, the model explicitly exploits both frequency-domain complementarity and hierarchical structural dependencies of texture representations, leading to more robust and discriminative feature learning for complex plant texture analysis tasks.

## 4 Conclusion

In this paper, we proposed a novel dual-domain hierarchical texture supervision network (DDHTS-Net) to address the challenges posed by the complex, diverse, and fine-grained nature of plant and general texture classification tasks. Recognizing the limitations of traditional single-network-based approaches in capturing the multi-scale and hierarchical characteristics inherent to texture patterns, DDHTS-Net introduces a multi-channel and multi-level supervision framework that integrates both spatial frequency decomposition and deep feature collaboration. Specifically, the Undecimated Wavelet Transform (UWT) is employed to construct four parallel channels with distinct granularity levels, enabling the network to separately extract local texture details and global structural information. To model the abstraction hierarchy of deep representations, an intra-channel hierarchical supervision unit is introduced, where higher-level experts guide lower-level ones based on feature differences, thus enhancing the learning of latent and critical texture attributes. Simultaneously, an inter-channel granularity-level supervision unit leverages differences among channels at the same abstraction level to guide the flow of cross-frequency knowledge within the original image channel, enabling more robust cross-domain feature modeling. Extensive experiments conducted on two plant classification datasets and two benchmark texture datasets demonstrate the superior performance and generalization ability of DDHTS-Net over existing state-of-the-art methods. These results validate the effectiveness of the proposed dual-domain supervision strategy and underscore the importance of multi-domain, multi-level feature integration in addressing the intrinsic challenges of texture analysis. Overall, DDHTS-Net provides a unified and interpretable framework for fine-grained texture feature extraction and classification, offering new insights and promising directions for future research in plant identification, material recognition, and broader texture analysis applications.

## Data Availability

Publicly available datasets were analyzed in this study. This data can be found here: PlantVillage Dataset is available at: https://www.kaggle.com/datasets/emmarex/plantdisease. Swedish Leaf Dataset is available at: https://www.cvl.isy.liu.se/en/research/datasets/swedish-leaf/. UIUC is available at: https://slazebni.cs.illinois.edu. UMD is available at: https://users.umiacs.umd.edu/~fer/High-resolutiondata-base/hr_database.htm.

## References

[B1] Abidin S. Togneri R. Sohel F. (2018). Spectrotemporal analysis using local binary pattern variants for acoustic scene classification. IEEE/ACM Trans. Audio Speech Lang. Process. 26, 2112–2121. doi: 10.1109/taslp.2018.2854861 25079929

[B2] Ali A. H. Youssef A. Abdelal M. Raja M. A. (2024). An ensemble of deep learning architectures for accurate plant disease classification. Ecol. Inform. 81, 102618. doi: 10.1016/j.ecoinf.2024.102618 38826717

[B3] Arivazhagan S. Ganesan L. (2003). Texture classification using wavelet transform. Pattern Recognit. Lett. 24, 1513–1521. doi: 10.1016/S0167-8655(02)00390-2

[B4] Atila Ü. Uçar M. Akyol K. Uçar E. (2021). Plant leaf disease classification using EfficientNet deep learning model. Ecol. Inform. 61, 101182. doi: 10.1016/j.ecoinf.2020.101182 38826717

[B5] Bell S. Upchurch P. Snavely N. Bala K. (2015). “ Material recognition in the wild with the materials in context database”, in: Proceedings of the IEEE conference on computer vision and pattern recognition, (Boston, MA: IEEE), 3479–3487. doi: 10.1109/CVPR.2015.7298970

[B6] Boudra S. Yahiaoui I. Behloul A. (2022). Tree trunk texture classification using multi-scale statistical macro binary patterns and CNN. Appl. Soft Comput. 118, 108473. doi: 10.1016/j.asoc.2022.108473 38826717

[B7] Chang M. Ji L. Zhu J. (2024). Multi-scale LBP fusion with the contours from deep CellNNs for texture classification. Expert Syst. Appl. 238, 122100. doi: 10.1016/j.eswa.2023.122100 38826717

[B8] Chen T. Yang W. Li S. Luo X. (2025). Data-driven calibration of industrial robots: a comprehensive survey. IEEE/CAA J. Autom. Sin. 12, 1544–1567. doi: 10.1109/JAS.2025.125237 25079929

[B9] Chen T. Yang W. Zhang Z. Luo X. (2024b). An efficient industrial robot calibrator with multiplaner constraints. IEEE Trans. Ind. Inform. 20, 14341–14350. doi: 10.1109/TII.2024.3450112 25079929

[B10] Chen Z. Quan Y. Xu R. Jin L. Xu Y. (2024a). Enhancing texture representation with deep tracing pattern encoding. Pattern Recognit. 146, 109959. doi: 10.1016/j.patcog.2023.109959 38826717

[B11] Cimpoi M. Maji S. Kokkinos I. Mohamed S. Vedaldi A. (2014). “ Describing textures in the wild”, in: Proceedings of the IEEE conference on computer vision and pattern recognition, (Columbus, OH: IEEE) 3606–3613. doi: 10.1109/CVPR.2014.461

[B12] Cimpoi M. Maji S. Vedaldi A. (2015). “ Deep filter banks for texture recognition and segmentation”, in: Proceedings of the IEEE conference on computer vision and pattern recognition, (Boston, MA: IEEE), 3828–3836. doi: 10.1109/CVPR.2015.7299007

[B13] Dai G. Tian Z. Fan J. Sunil C. K. Dewi C. (2024). DFN-PSAN: Multi-level deep information feature fusion extraction network for interpretable plant disease classification. Comput. Electron. Agric. 216, 108481. doi: 10.1016/j.compag.2023.108481 38826717

[B14] Dong X. Zhou H. Dong J. (2020). Texture classification using pair-wise difference pooling-based bilinear convolutional neural networks. IEEE Trans. Image Process. 29, 8776–8790. doi: 10.1109/TIP.2020.3019185 32866099

[B15] Du R. Chang D. Bhunia A. K. Xie J. Ma Z. Song Y. Z. . (2020). “ Fine-grained visual classification via progressive multi-granularity training of jigsaw patches”, in: European conference on computer vision, (Cham: Springer), 153–168. doi: 10.1007/978-3-030-58565-5_10

[B16] Florindo J. B. (2020). DSTNet: Successive applications of the discrete Schroedinger transform for texture recognition. Inf. Sci. 507, 356–364. doi: 10.1016/j.ins.2019.08.049 38826717

[B17] Florindo J. B. Backes A. R. Neckel A. (2024). ELMP-Net: The successive application of a randomized local transform for texture classification. Pattern Recognit. 153, 110499. doi: 10.1016/j.patcog.2024.110499 38826717

[B18] Florindo J. Bruno O. M. (2022). “ Using fractal interpolation over complex network modeling of deep texture representation”, in: 2022 Eleventh International Conference on Image Processing Theory, Tools and Applications (IPTA), (Salzburg: IEEE), 1–5. doi: 10.1109/IPTA54936.2022.9784138

[B19] Florindo J. B. Lee Y. S. Jun K. Jeon G. Albertini M. K. (2021). VisGraphNet: A complex network interpretation of convolutional neural features. Inf. Sci. 543, 296–308. doi: 10.1016/j.ins.2020.07.050 38826717

[B20] Fritz S. See L. Bayas J. C. L. Waldner F. Jacques D. Becker-Reshef I. . (2019). A comparison of global agricultural monitoring systems and current gaps. Agric. Syst. 168, 258–272. doi: 10.1016/j.agsy.2018.05.010 38826717

[B21] Geetharamani G. Pandian A. (2019). Identification of plant leaf diseases using a nine-layer deep convolutional neural network. Comput. Electr. Eng. 76, 323–338. doi: 10.1016/j.compeleceng.2019.04.011 38826717

[B22] Ghalati M. K. Nunes A. Ferreira H. Serranho P. Bernardes R. (2021). Texture analysis and its applications in biomedical imaging: A survey. IEEE Rev. Biomed. Eng. 15, 222–246. doi: 10.1109/RBME.2021.3115703 34570709

[B23] Gokulnath B. V. (2021). Identifying and classifying plant disease using resilient LF-CNN. Ecol. Inform. 63, 101283. doi: 10.1016/j.ecoinf.2021.101283 38826717

[B24] Gu J. Wang Z. Kuen J. Ma L. Shahroudy A. Shuai B. . (2018). Recent advances in convolutional neural networks. Pattern Recognit. 77, 354–377. doi: 10.1016/j.patcog.2017.10.013 38826717

[B25] Hadid A. Ylioinas J. Bengherabi M. Ghahramani M. Taleb-Ahmed A. (2015). Gender and texture classification: A comparative analysis using 13 variants of local binary patterns. Pattern Recognit. Lett. 68, 231–238. doi: 10.1016/j.patrec.2015.04.017 38826717

[B26] Hanh B. T. Van Manh H. Nguyen N. V. (2022). Enhancing the performance of transferred efficientnet models in leaf image-based plant disease classification. J. Plant Dis. Prot.129. 3, 623–634. doi: 10.1007/s41348-022-00601-y 30311153

[B27] He D. C. Wang L. (1990). Texture unit, texture spectrum, and texture analysis. IEEE Trans. Geosci. Remote Sens. 28, 509–512. doi: 10.1109/IGARSS.1989.575836 25079929

[B28] Hu Y. Liu X. Zhang B. Han J. Cao X. (2021). Alignment enhancement network for fine-grained visual categorization. ACM Trans. Multim. Comput. Commun. Appl. 17, 1–20. doi: 10.1109/CVPR.2016.132 25079929

[B29] Huang D. Shan C. Ardabilian M. Wang Y. Chen L. (2011). Local binary patterns and its application to facial image analysis: a survey. IEEE Trans. Syst. Man Cybern. C. Appl. Rev. 41, 765–781. doi: 10.1109/TSMCC.2011.2118750 25079929

[B30] Hughes D. Salathé M. (2015). An open access repository of images on plant health to enable the development of mobile disease diagnostics. Arxiv Preprint Arxiv:1511.08060. doi: 10.48550/arXiv.1511.08060

[B31] Kamal K. C. Yin Z. Wu M. Wu Z. (2019). Depthwise separable convolution architectures for plant disease classification. Comput. Electron. Agric. 165, 104948. doi: 10.1016/j.compag.2019.104948 38826717

[B32] Kaya Y. Gürsoy E. (2023). A novel multi-head CNN design to identify plant diseases using the fusion of RGB images. Eco. Inform. 75, 101998. doi: 10.1016/j.ecoinf.2023.101998 38826717

[B33] Krizhevsky A. Sutskever I. Hinton G. E. (2012). Imagenet classification with deep convolutional neural networks. Adv. Neural Inf. Process. Syst. 25, 1097–1105. doi: 10.5555/2999134.2999257

[B34] Lazebnik S. Schmid C. Ponce J. (2005). A sparse texture representation using local affine regions. IEEE Trans. Pattern Anal. Mach. Intell. 27, 1265–1278. doi: 10.1109/TPAMI.2005.151 16119265

[B35] Lee S. H. Chan C. S. Mayo S. J. Remagnino P. (2017). How deep learning extracts and learns leaf features for plant classification. Pattern Recognit. 71, 1–13. doi: 10.1016/j.patcog.2017.05.015 38826717

[B36] Li Z. Li S. Bamasag O. O. Alhothali A. Luo X. (2022b). Diversified regularization enhanced training for effective manipulator calibration. IEEE Trans. Neural Netw. Learn. Syst. 34, 8778–8790. doi: 10.1109/TNNLS.2022.3153039 35263261

[B37] Li Z. Li S. Francis A. Luo X. (2022a). A novel calibration system for robot arm via an open dataset and a learning perspective. IEEE Trans. Circuits Syst. II Express Briefs 69, 5169–5173. doi: 10.1109/TCSII.2022.3199158 25079929

[B38] Li Z. Liu F. Yang W. Peng S. Zhou J. (2021). A survey of convolutional neural networks: analysis, applications, and prospects. IEEE Trans. Neural Netw. Learn. Syst. 33, 6999–7019. doi: 10.1109/TNNLS.2021.3084827 34111009

[B39] Lima G. V. L. D. Saito P. T. M. Lopes F. M. Bugatti P. H. (2019). Classification of texture based on bag-of-visual-words through complex networks. Expert Syst. Appl. 133, 215–224. doi: 10.1016/j.eswa.2019.05.021 38826717

[B40] Liu L. Chen J. Zhao G. Fieguth P. Chen X. Pietikäinen M. (2019). Texture classification in extreme scale variations using GANet. IEEE Trans. Image Process. 28, 3910–3922. doi: 10.1109/TIP.2019.2903300 30869616

[B41] Mahapatra P. Panda M. Dash S. K. Sahu U. K. (2026). Advancing plant disease classification using an attention-based CNN for intra-dataset and cross-dataset training. Sci. Rep. 16, 10925. doi: 10.1038/s41598-026-45464-7 41896662 PMC13039305

[B42] Mao S. Rajan D. Chia L. T. (2021). Deep residual pooling network for texture recognition. Pattern Recognit. 112, 107817. doi: 10.1016/j.patcog.2021.107817 38826717

[B43] Mohan A. Peeples J. (2024). “ Lacunarity pooling layers for plant image classification using texture analysis”, in: In proceedings of the IEEE/CVF conference on computer vision and pattern recognition, (Seattle, WA: IEEE), 5384–5392. doi: 10.1109/CVPRW63382.2024.00547

[B44] Moupojou E. Tagne A. Retraint F. Tadonkemwa A. Wilfried D. Tapamo H. . (2023). FieldPlant: A dataset of field plant images for plant disease detection and classification with deep learning. IEEE Access 11, 35398–35410. doi: 10.1109/ACCESS.2023.3263042 25079929

[B45] Ojala T. Pietikainen M. Maenpaa T. (2002). Multiresolution gray-scale and rotation invariant texture classification with local binary patterns. IEEE Trans. Pattern Anal. Mach. Intell. 24, 971–987. doi: 10.1109/TPAMI.2002.1017623 25079929

[B46] Park S. H. Ahn S. Y. Lee S. W. (2024). Deep feature retention module network for texture classification. Appl. Sci. 14, 4011. doi: 10.3390/app14104011 30654563

[B47] Paymode A. S. Malode V. B. (2022). Transfer learning for multi-crop leaf disease image classification using convolutional neural network VGG. Artif. Intell. Agric. 6, 23–33. doi: 10.1016/j.aiia.2021.12.002 38826717

[B48] Peng J. Huang Y. Sun W. Chen N. Ning Y. Du Q. (2022). Domain adaptation in remote sensing image classification: A survey. IEEE J. Sel. Top. Appl. Earth Obs. Remote Sens. 15, 9842–9859. doi: 10.1109/JSTARS.2022.3220875 25079929

[B49] Russel N. S. Selvaraj A. (2022). Leaf species and disease classification using multiscale parallel deep CNN architecture. Neural Comput. Appl. 34, 19217–19237. doi: 10.1007/s00521-022-07521-w 30311153

[B50] Sakthipriya G. Padmapriya N. Venkateswaran N. (2024). TSDAnet: texture strip dual attention network for intraclass texture classification. Signal. Image Video Process. 18, 7597–7610. doi: 10.1007/s11760-024-03413-9 30311153

[B51] Scabini L. F. Ribas L. C. Bruno O. M. (2020). Spatio-spectral networks for color-texture analysis. Inf. Sci. 515, 64–79. doi: 10.1016/j.ins.2019.11.042 38826717

[B52] Shafik W. Tufail A. De Silva Liyanage C. Apong R. A. A. H. M. (2024). Using transfer learning-based plant disease classification and detection for sustainable agriculture. BMC Plant Biol. 24, 136. doi: 10.1186/s12870-024-04825-y 38408925 PMC10895770

[B53] Shah M. P. Singha S. Awate S. P. (2017). “ Leaf classification using marginalized shape context and shape+ texture dual-path deep convolutional neural network”, in: 2017 IEEE International conference on image processing (ICIP), (Beijing: IEEE), 860–864. doi: 10.1109/ICIP.2017.8296403

[B54] Simonyan K. Zisserman A. (2014). “ Very deep convolutional networks for large-scale image recognition,” in Arxiv Preprint Arxiv:1409.1556. doi: 10.48550/arXiv.1409.1556

[B55] Singh C. B. Choudhary R. Jayas D. S. Paliwal J. (2010). Wavelet analysis of signals in agriculture and food quality inspection. Food. Bioproc. Technol. 3, 2–12. doi: 10.1007/s11947-008-0093-7 30311153

[B56] Söderkvist O. (2001). Computer vision classification of leaves from swedish trees. M aster’s thesis, Linköping University, SE-581 83 Linköping, Sweden. LiTH-ISY-EX-3132.

[B57] Starck J. L. Fadili J. Murtagh F. (2007). The undecimated wavelet decomposition and its reconstruction. IEEE Trans. Image Process. 16, 297–309. doi: 10.1109/TIP.2006.887733 17269625

[B58] Syed S. A. Iqbal M. Z. Riaz M. M. (2017). Describing contrast across scales. ISPRS J. Photogramm. Remote Sens. 128, 326–337. doi: 10.1016/j.isprsjprs.2017.04.002 38826717

[B59] Tao Z. Wei T. Li J. (2021). Wavelet multi-level attention capsule network for texture classification. IEEE Signal Process Lett. 28, 1215–1219. doi: 10.1109/LSP.2021.3088052 25079929

[B60] Thakur P. S. Sheorey T. Ojha A. (2023). VGG-ICNN: A lightweight CNN model for crop disease identification. Multimed. Tools Appl. 82, 497–520. doi: 10.1007/s11042-022-13144-z 30311153

[B61] Valarmathi G. Suganthi S. U. Subashini V. Janaki R. Sivasankari R. Dhanasekar S. (2021). CNN algorithm for plant classification in deep learning. Mater. Today Proc. 46, 3684–3689. doi: 10.1016/j.matpr.2021.01.847 38826717

[B62] Wang W. Jiang Y. Wang G. Guo F. Li Z. Liu B. (2022). Multi-scale LBP texture feature learning network for remote sensing interpretation of land desertification. Remote Sens. 14, 3486. doi: 10.3390/rs14143486 30654563

[B63] Wang B. Wang D. (2019). Plant leaves classification: A few-shot learning method based on siamese network. IEEE Access 7, 151754–151763. doi: 10.1109/ACCESS.2019.2947510 25079929

[B64] Wu H. Fang L. Yu Q. Yuan J. Yang C. (2023). Plant leaf identification based on shape and convolutional features. Expert Syst. Appl. 219, 119626. doi: 10.1016/j.eswa.2023.119626 38826717

[B65] Xu Y. Ji H. Fermüller C. (2009). Viewpoint invariant texture description using fractal analysis. Int. J. Comput. Vis. 83, 85–100. doi: 10.1007/s11263-009-0220-6 30311153

[B66] Xu X. Li B. Wu Q. J. (2025). Dual-stream multi-layer cross encoding network for texture analysis of architectural heritage elements. NPJ Heritage Sci. 13, 493. doi: 10.1038/s40494-025-02066-2 37880705

[B67] Yang C. (2021). Plant leaf recognition by integrating shape and texture features. Pattern Recognit. 112, 107809. doi: 10.1016/j.patcog.2020.107809 38826717

[B68] Zhai W. Cao Y. Zha Z. J. Xie H. Wu F. (2020). “ Deep structure-revealed network for texture recognition”, in: Proceedings of the IEEE/CVF Conference on Computer Vision and Pattern Recognition, 11010–11019. doi: 10.1109/CVPR42600.2020.01102

[B69] Zhang X. Yan L. Abuhaija B. Ihnaini B. (2025). MCP-X: An ultra-compact CNN for rice disease classification in resource-constrained environments. AgriEngineering 7, 359. doi: 10.3390/agriengineering7110359 30654563

[B70] Zou C. Wang R. Jin C. Zhang S. Wang X. (2023). S2CL-Leaf Net: Recognizing leaf images like human botanists. ACM Trans. Multimed. Comput. Commun. Appl. 20, 1–2. doi: 10.1145/3615659

